# Polyphenols as Drivers of a Homeostatic Gut Microecology and Immuno-Metabolic Traits of *Akkermansia muciniphila*: From Mouse to Man

**DOI:** 10.3390/ijms24010045

**Published:** 2022-12-20

**Authors:** María Carolina Rodríguez-Daza, Willem M. de Vos

**Affiliations:** 1 Laboratory of Microbiology, Wageningen University & Research, Stippeneng 4, 6708 WE Wageningen, The Netherlands; 2 Human Microbiome Research Program, Faculty of Medicine, University of Helsinki, 00014 Helsinki, Finland

**Keywords:** *Akkermansia muciniphila*, gut microbiota, polyphenols, ecological niche, AhR, IL-22, polyphenol degraders, phenolic metabolites

## Abstract

*Akkermansia muciniphila* is a mucosal symbiont considered a gut microbial marker in healthy individuals, as its relative abundance is significantly reduced in subjects with gut inflammation and metabolic disturbances. Dietary polyphenols can distinctly stimulate the relative abundance of *A. muciniphila*, contributing to the attenuation of several diseases, including obesity, type 2 diabetes, inflammatory bowel diseases, and liver damage. However, mechanistic insight into how polyphenols stimulate *A. muciniphila* or its activity is limited. This review focuses on dietary interventions in rodents and humans and in vitro studies using different phenolic classes. We provide critical insights with respect to potential mechanisms explaining the effects of polyphenols affecting *A. muciniphila*. Anthocyanins, flavan-3-ols, flavonols, flavanones, stilbenes, and phenolic acids are shown to increase relative *A. muciniphila* levels in vivo, whereas lignans exert the opposite effect. Clinical trials show consistent findings, and high intervariability relying on the gut microbiota composition at the baseline and the presence of multiple polyphenol degraders appear to be cardinal determinants in inducing *A. muciniphila* and associated benefits by polyphenol intake. Polyphenols signal to the AhR receptor and impact the relative abundance of *A. muciniphila* in a direct and indirect fashion, resulting in the restoration of intestinal epithelial integrity and homeostatic crosstalk with the gut microbiota by affecting IL-22 production. Moreover, recent evidence suggests that *A. muciniphila* participates in the initial hydrolysis of some polyphenols but does not participate in their complete metabolism. In conclusion, the consumption of polyphenol-rich foods targeting *A. muciniphila* as a pivotal intermediary represents a promising precision nutritional therapy to prevent and attenuate metabolic and inflammatory diseases.

## 1. Introduction

The human gut houses trillions of microbes, including viruses, fungi, archaea, and bacteria, the latter being the major representative [1,2]. The gut microbiota plays an essential role in human health. Deviations in the composition and functions of the gut microbiota have been associated with many diseases, including gastrointestinal, cardiovascular [3,4,5,6], hepatic [7], immunological, and neurological disorders [8,9].

**Figure 1:**
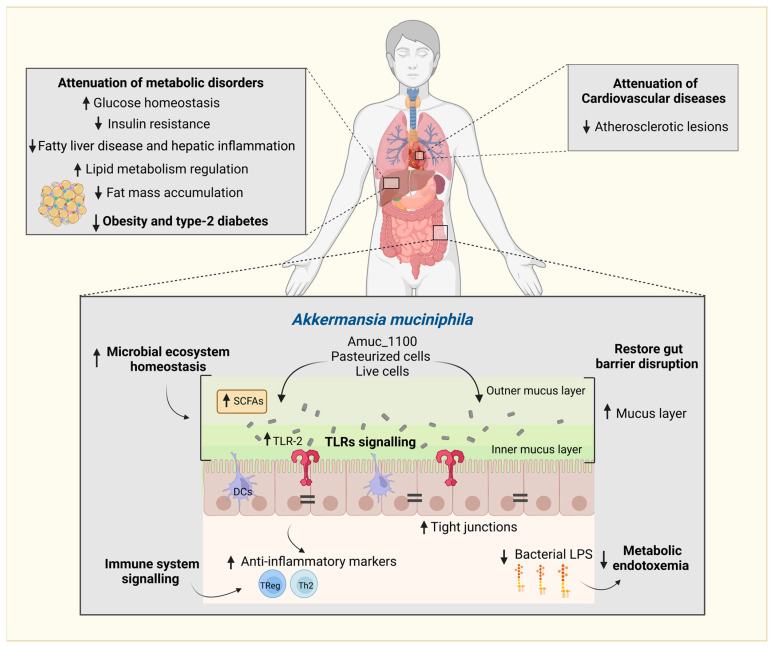
Beneficial impact of *Akkermansia muciniphila* on host health. *A. muciniphila* protects against cardiovascular diseases, metabolic disorders, and intestinal microecological disruption. An increased relative proportion of *A. muciniphila* in the gut microbiota has been inversely associated with obesity and type 2 diabetes. The administration of active *A. muciniphila*; its membrane protein, Amuc_1100; and pasteurized cells can reverse metabolic disorders associated with a high-fat diet, obesity, and type 2 diabetes. Clinical and preclinical studies highlight the role of *A. muciniphila* in attenuating fat mass accumulation, adipose tissue inflammation, insulin resistance, and glucose homeostasis. *A. muciniphila* alleviates chronic intestinal inflammation and metabolic endotoxemia (reduces the level of lipopolysaccharide (LPS)) and induces immune cell and Toll-like receptor (TLR)-2 signaling. Owing to its glycan-degrading capabilities and proximity to the host colonic epithelium, this bacterium contributes to the maintenance of the mucus layer barrier function and intestinal homeostasis. The graph was created with BioRender.

A. *muciniphila*, a member of the phylum Verrucomicrobia, is an anaerobic and mucosa-associated colonic bacterium with mucin-degrading capabilities that has been identified as a microbial marker of a homeostatic intestine [10]. It has been linked to potential benefits by counteracting gut barrier disruption, obesity, and related metabolic disturbances [10,11,12,13]. Recently in humans [11] and rodents [14,15,16], treatment with *A. muciniphila* was shown to enhance obesity and related disorders, including glucose tolerance, insulin resistance, fat mass gain, total plasma cholesterol, liver dysfunction, and low-grade inflammation (Figure 1). A specific protein present on the outer membrane of this bacterium, termed Amuc_1100, has been demonstrated to be implicated in the interaction with the host mucosa via toll-like receptor-2 (TLR2) signaling, restoring the intestinal barrier function and decreasing metabolic endotoxemia in mice [10,14,17].

Obesity, type 2 diabetes (T2D), and chronic consumption of a high-fat diet (HFD) have detrimental effects on the gut microbiota composition, especially on relative levels of *A. muciniphila,* altering the integrity of the intestinal epithelium and symbiotic host–microbe crosstalk [11,18,19,20,21]. For instance, a long-term HFD leads to mucin (Muc2) protein misfolding and endoplasmic reticulum (ER) stress and reduces Muc2 production and O-glycosylation by goblet cells (GCs) [22,23,24]. Hence, the accumulation of non-glycosylated Muc2 precursors weakens the protective mucus layer and exacerbates the adhesion and mucus degradation by opportunistic pathogens, in addition to reducing mucosa-associated symbionts, such as *A. muciniphila* [24,25]. These gut ecological disturbances were reported to notably reduce the *A. muciniphila* population but were shown to be prevented and restored by the consumption of polyphenols [26,27].

Polyphenols are chemically diverse molecules synthesized by plants as secondary metabolites. They exert pharmacological properties, such as antioxidant, anticarcinogenic, antimicrobial, and anti-inflammatory activities [28,29,30,31,32]. Most of the bioactivity of polyphenols is thought to be mediated by their interaction with the host gut microbiota. Indeed, several reports have demonstrated the poor bioavailability of polyphenols by using cellular cultures mimicking the epithelium of the small and large intestines or by measuring the metabolites from gut microbial degradation of these compounds in vivo [33,34,35,36]. Accumulating studies suggest that dietary polyphenols may stimulate the growth of *A. muciniphila* in vivo and show this bacterium to be a critical player boosting the bioactivities of polyphenols in different health conditions, including health-steady status, cardiovascular diseases, liver damage, and gut inflammation [37,38,39,40,41,42,43,44]. However, the basic mechanisms of the potential prebiotic effects of polyphenols on *A. muciniphila* are unclear.

Distinct mechanisms might be implicated in the promoting effects of polyphenols on *A. muciniphila*. Polyphenols can restore gut microbiota disbalance and mucus barrier depletion in obesity and inflammatory bowel diseases (IBD) [44,45]. In the colon, these molecules interact with more than 1000 species of bacteria that might have the catalytic and hydrolytic ability to metabolize polyphenols to the corresponding aglycone and derived metabolites [36,46]. On the one hand, phenolics and derivatives can act as antimicrobials, inhibiting opportunistic bacteria that are abundant in metabolic diseases [28]. On the other hand, polyphenols and phenolic metabolites can simultaneously act locally in the intestinal mucosa, be absorbed through the epithelium into the systemic circulation, and regulate anti-inflammatory markers [27,28,29,30]. In line with this, the activation of the aryl hydrocarbon receptor (*AhR*) and the induction of interleukin (IL)-22 appear to be involved in the mechanisms of action, leading to a protective O-glycan-rich mucus layer and reduced mucosal inflammation by polyphenols [47,48,49]. However, in this context, it should be mentioned that *A. muciniphila* can also directly induce IL-22, as shown recently [50]. Recent studies indicate that polyphenols induce goblet cell (GC) differentiation, increase mucus layer thickness, and reduce colitis. However, it remains unclear whether this is a direct effect or whether *A. muciniphila* is involved [26,27,51].

The strong association between the consumption of polyphenols and the relative abundance of *A. muciniphila* in humans and animals also suggests the trophic utilization of phenolics by this bacterium. Indeed, individuals with a polyphenol-degrading metabotype that yields the production of phenolic metabolites, such as urolithin A, present with an increased relative abundance of *A. muciniphila* and a better metabolic outcome and health status than those lacking a specific polyphenol-degrading species [43,52,53]. Several enzymes have been identified to participate in the polyphenol metabolism by gut bacteria [44]. However, none has been described and tested to date in *A. muciniphila*. A few in vitro studies on the growth of *A. muciniphila* in polyphenol-enriched media suggest the nutritional utilization of phenolics [43,54]. However, diverging findings on the produced metabolites and the reduced growth of *A. muciniphila* in the presence of polyphenols as a unique nutritional source or combined with mucin and glucose prevent the confirmation of such a hypothesis.

The aim of the present review is to provide an overview of polyphenols that shape the composition of the gut microbiota, particularly promoting the relative abundance of *A. muciniphila*, with a protective role against metabolic and inflammatory disorders. We identified compelling trials in rodents and humans in *A. muciniphila* was shown to be selectively induced by different classes of dietary polyphenols. We focused on the impact of polyphenols and derived metabolites on the mucosal immunomodulation and re-establishment of the gut microenvironment, improving the ecological niche of *A. muciniphila* as a potential non-prebiotic mechanism. Additionally, hints with respect to the metabolism of polyphenols by *A.* *muciniphila* and its possible molecular adaptation to these compounds are discussed. Preclinical and clinical dietary interventions demonstrate that *A. muciniphila* potentiates many polyphenol-induced processes by regulating host metabolism and inflammation, contributing to the maintenance of gut homeostasis.

## 2. *Akkermansia muciniphila*, the Gut Microenvironment, and Immune Regulation

The intestinal epithelial barrier is enhanced by the presence of a mucus layer and immune factors produced by the host [55]. This mucus layer secreted by GCs consists mainly of a mesh-like network of gel-forming Muc2 mucin, with tandem repeated sequences rich in threonine and serine residues acting as the attachment sites of O-glycans [56]. These O-glycans primarily consist of N-acetylgalactosamine, N-acetylglucosamine, fucose, galactose, sialic acid (N-acetylneuraminic acid), mannose, and sulfate. The post-translational O-glycosylation provides resistance to proteolysis by traditional proteases, furnishes selective commensal microbes, and prevents pathogen colonization [25]. *A. muciniphila* is a prominent O-glycan utilizer species capable of hydrolyzing glycosidic linkages and degrading the colonic mucin. Its genome is equipped with specialized glycosyl hydrolases, including α-fucosidases, α-sialidases, and endo-α-N-acetylgalactosaminidases [57,58]. All these features give *A. muciniphila* the selective trophic advantage to prevail, signal immunological response in the intestinal mucosa, and contribute to regular mucus turnover. Its relative abundance positively correlates with mucus layer thickness, intestinal integrity, reduced endotoxemia and intestinal inflammation, and improved metabolic phenotype in humans and animals (Figure 1) [10,11,59]. Mechanistic studies conducted in vitro and in rodent models demonstrate that *A. muciniphila* increases transepithelial electrical resistance; stimulates GCs and mucin secretion; alleviates chronic intestinal inflammation; regulates glucose and lipid metabolism; and induces immune responses, including T-cell receptors, B-cell receptors, NF-κB activation, and TLR2 signaling [14,17,59,60].

The structure and glycosylation pattern of mucin are critical modulators of the gut microbiome, particularly *A. muciniphila*. *O*-glycosylation defects drive a disrupted niche in the intestinal mucosa and relate to the onset of gut inflammation and mucin binding and degradation by microbial pathogens. For instance, minimal sulfate modification and a reduced abundance of *A. muciniphila* have been reported in subjects with ulcerative colitis [61,62,63]. A reduced proportion of fucosylated glycans and an increased proportion of hyposialylated T-antigen glycans have been associated with exacerbated inflammation [61,64]. Interestingly, such abnormalities are attenuated by the exogenous administration of mucin O-glycans, modulating gut microbiota alterations, notably by inhibiting pathogens and favoring *A. muciniphila* [65].

The regulation of gut immunity and host glycans is fundamental to maintain a protective gut mucosal barrier. Signals regulating the innate immune response, such as induction of the aryl hydrocarbon receptor (*AhR*), have been shown to bridge homeostasis between the gut microbiota and the intestinal epithelium. The ligand-activated *AhR* leads to the transcription of several genes, such as *Rort* and *Foxp3,* and modulates interleukin (IL)-22 secretion, with a pivotal role in mucosal defense [66]. For instance, *IL-22* can induce fucosylation of intestinal epithelial cells and prevent the overcolonization of opportunistic pathogens [67]. The role of these markers in signaling colonic innate response makes them essential regulators of the gut mucus barrier and the colonization of *A. muciniphila*. The *AhR* senses dietary compounds and microbial metabolites, including those of tryptophan metabolism. The modulation of the gut microbiota, especially the stimulation of tryptophan-metabolizing species, has been associated with the enrichment of *AhR* ligands and improved mucosal microenvironment [68,69]. Recently, *A. muciniphila* was also shown to metabolize tryptophan to indole derivatives and *AhR* agonists [70], in addition to being found to induce IL22 in a MyD88-dependent fashion in mice [50]. Similarly, the live and pasteurized cells of *A. muciniphila* and its membrane protein, Amuc_1100, were shown to induce *AhR*-targeted genes, including *CYP1A1*, *IL-10*, and *IL-22*, protecting against ulcerative colitis in mice [71]. Additionally, integrative analysis of fecal metagenomes and untargeted serum metabolomes from patients and mice with ulcerative colitis highlighted a significant correlation between *A. muciniphila* and the restoration of tryptophan metabolism [71]. A synergic effect might exist between *AhR* modulators and the abundance of *A. muciniphila* in triggering gut immune regulation by shapes on *AhR* signaling, thereby attenuating colonic inflammation and metabolic alterations.

The increased abundance of *A. muciniphila* might be sustained by the polyphenol-driven restoration of mucosal integrity. However, it is not possible to exclude the crosstalk between polyphenols and the gut microbiota from the mechanisms underpinning the stimulation of *A. muciniphila*. The sections below tackle the role of different polyphenol classes as modulators of gut microbiota composition, especially *A. muciniphila.* Furthermore, mechanistic insights into the effects of polyphenols in regulating immune markers linked to the attenuation of gut inflammation and improved intestinal barrier function are discussed.

## 3. Interactions between Polyphenols and the Gut Microbiota and Their Impact on the Attenuation of Chronic Diseases

**Figure 2:**
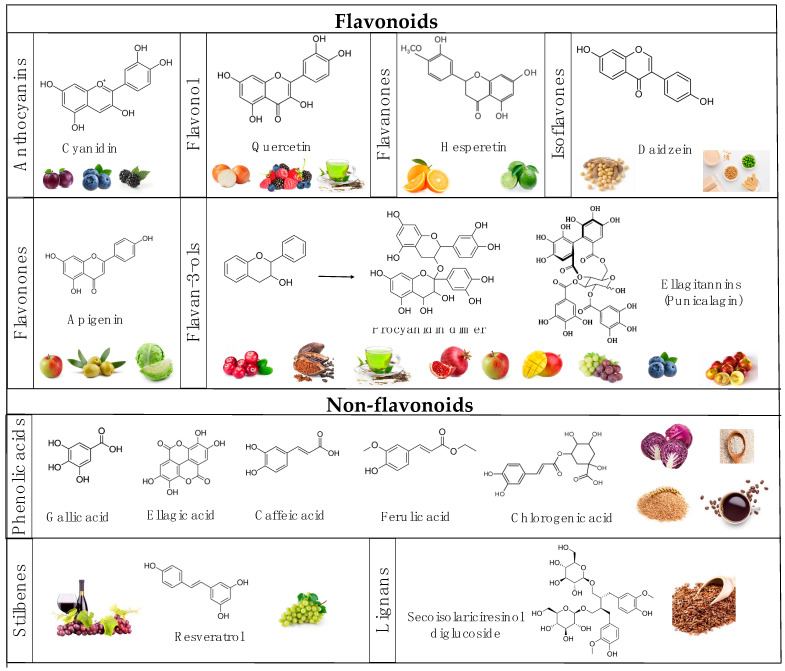
Chemical structures of polyphenol classes of flavonoids and non-flavonoids and their main food sources.

Polyphenols are categorized into two main structural classes—flavonoids and non-flavonoids—which vary in the number of phenolic rings and their type of linkages, as well as the main dietary sources (Figure 2). The flavonoid group is subcategorized into anthocyanins, flavonols, flavanones, isoflavones, and flavan-3-ols. The non-flavonoid group includes phenolic acids, stilbenes, and lignans [44]. Vegetables and fruits, such as pomegranates, berries, grapes, tea, cocoa, and coffee, are rich sources of polyphenols.

Owing to the poor biological availability of dietary polyphenols [72], growing evidence highlights the gut microbiota as a critical intermediary involved in priming host cell signaling in the intestinal mucosa and distal organs; these interactions are associated with diverse biological roles in preventing and attenuating the chronicity of several diseases [31,73,74,75,76,77,78,79]. Approximately 90–95% of polyphenol intake accumulates in the intestinal lumen up to the millimolar range, where, together with bile conjugates, it is subjected to the enzymatic activities by the gut microbiota, sequentially producing metabolites with different physiological significance [35]. Ottaviani et al. (2016) [36] evaluated the pharmacokinetic profile of polyphenol metabolism in humans, outlining the distinguished absorption of different phenolic compounds in the proximal gastrointestinal tract and the colon. The authors assessed the absorption, distribution, metabolism, and excretion of (−)-epicatechin (EC), flavanol monomers, and procyanidins, with a degree of polymerization (DP) of 2–10. Based on the timing of the appearance of the various radiolabeled phenolic compounds in plasma and urine, it was demonstrated that most EC and procyanidin (EC polymer) absorption occurred in the colon. For instance, EC-derived metabolites were absent from plasma after 6–8 h, indicating that the ingested EC and polymers continued down the gastrointestinal tract and reached the colon. In the colon, gut microbiota–polyphenol interactions, which start with the C-ring opening, yielded the formation of O-glucuronidated and sulfated derivatives of phenyl- γ-valerolactones (γVL). Here, more than 70% of the ingested, radiolabeled (−)epicatechin flavanols were shown to be absorbed into the circulatory system via the colon, compared to 20% absorbed from the small intestine. The phenolic metabolites were further submitted to phase II metabolism by enzymes in the colon mucosa and the liver, resulting in increased levels of a mixture of γVLs and phenolic acids in the blood circulation [36]. This provides insights into the role of the gut microbiome in generating phenolic bioactive metabolites in the human body. Many of these metabolic outputs are involved in various physiological processes, as reviewed by Man and colleagues (2020) [80].

For instance, the biological roles of parent phenolic compounds and the de novo generated bioactive molecules by the gut microbiota have been demonstrated in the regulation of inflammation [74], oxidative stress, [75], thermogenesis [31], apoptosis; proliferation of cancer cells [76]; blood pressure regulation; mitochondrial functions; and glucose and lipid metabolism [77,78,79].

Polyphenols exert beneficial effects on the gut microbiota by acting as prebiotics. In 2017, the ISAPP (International Scientific Association for Probiotics and Prebiotics) updated the prebiotic concept by considering phenolics/phytochemicals between the potential prebiotic candidates acting similarly as prebiotics. To fit in this category, phenolics must be selectively utilized by host microbes, conferring a health benefit [81]. The stimulation of beneficial microbes is related to their enzymatic capacity and adaptation to degrade and tolerate phenolics with different structural features. Increased attention is given to the stimulation of beneficial species by polyphenol intake in humans and animals, especially *A. muciniphila,* as a promising dietary strategy to counteract obesity and diabetes (table). *A. muciniphila* is relatively abundant in healthy individuals, accounting for approximately 4% of the gut microbiota. However, its abundance declines significantly in certain metabolic diseases [11,14,60].

*A. muciniphila* appears to be essential in boosting polyphenol-mediated effects to cope with obesity. Specifically, this species has been correlated with higher levels of a phenolic metabolite, urolithin A, and polyphenol-degrader species, such as *Gordonibacter* spp. [53]. Although a gap in the enzymatic ability of *A. muciniphila* to degrade polyphenols is yet to be filled, this species is regularly implicated in immune–metabolic processes promoted by dietary polyphenols in preclinical, in vitro, and clinical trials. Here, we compiled major findings on the effects of polyphenol-rich foods on the modulation of *A. muciniphila* abundance in the context of several chronic diseases.

## 4. Modulation of *A. muciniphila* by Polyphenol-Rich Foods In Vitro and in Animal Models

### 4.1. Anthocyanin-Rich Foods

**Figure 3:**
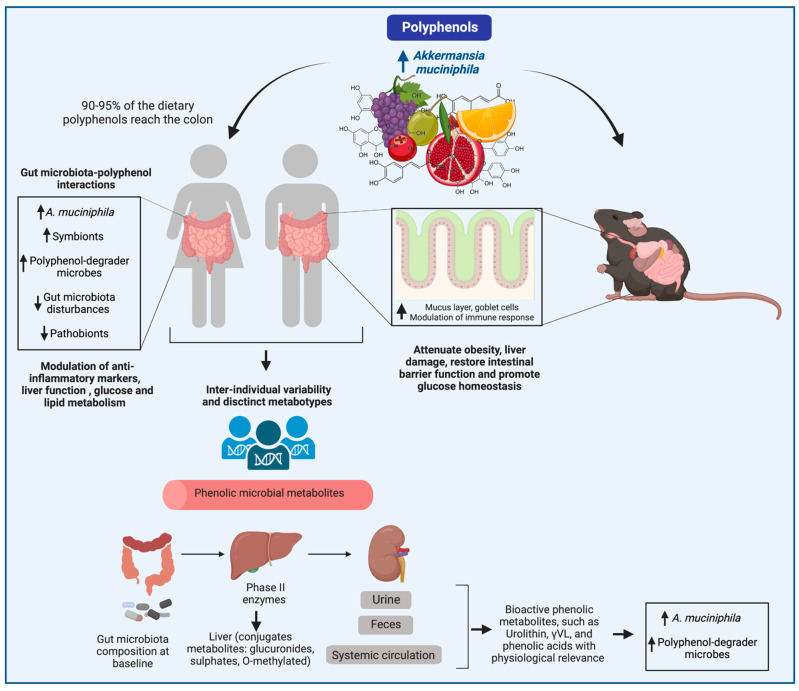
Schematic illustration of the modulatory effects of polyphenols on the gut microbiota, especially on *A. muciniphila* in humans and animals. A vast proportion of dietary polyphenols reach the colon, interacting with the gut microbiota. Polyphenols reduce gut microbiota disturbances and promote the relative abundance of *A. muciniphila* in humans and rodents. These changes are associated with improved metabolic phenotypes in the host. Interindividual variability in humans influences the effects of polyphenols on the gut microbiota and the production of bioactive metabolites, such as phenyl-γ-valerolactones (γVL), urolithins, and phenolic acids. Mechanistic insights into animal models sustain the role of polyphenols in signaling anti-inflammatory immune markers, increasing mucin-secreting goblet cells, and restoring intestinal barrier function. The graph was created with BioRender.

Anthocyanins are glycosides of anthocyanidins, one of the major flavonoids widely found in berries. Plum, beans, and pomegranate are also good sources of anthocyanins. The identified anthocyanins are cyanidin, malvidin, petunidin, pelargonidin, peonidin, and delphinidin [82]. These compounds are mainly described as poorly available in the small intestine and have been extensively studied due to their potent antioxidant properties and benefits on human health [83] (Figure 3). The conversion of anthocyanins by gut bacteria is related to their β-glucosidase enzymatic capability [84]. Bacterial species such as *Lantiplantibacillus plantarum*, *Lactobacillus acidophilus, Streptococcus thermophiles, Lactobacillus delbrueckii* subsp. *bulgaricus, Bacteroides thetaiotaomicron*, *Blautia producta, Erysipelatoclostridium ramosum*, *Bifidobacterium animalis,* and *Bifidobacterium* spp. are involved in degrading anthocyanins [84,85].

The intake of anthocyanin-rich fruits has been shown to stimulate *A. muciniphila* and attenuate host gut inflammation, liver injury, and obesity-associated metabolic alterations (Table 1). Recently, Song and colleagues (2020) investigated whether anthocyanins play a crucial role in the antidiabetic effect of açai fruits (*Euterpe oleracea* Mart.) in high-fat diet (HFD)-induced obesity in mice [86]. The anthocyanin-rich açai extract significantly shifted the overall structure and composition of the gut microbiota, in addition to ameliorating HFD-induced glucose intolerance and insulin resistance. The relative abundance of *A. muciniphila* was mainly induced by the açai extract and highlighted as a key player driving the anthocyanin-induced antiobesity effects. Increased *A. muciniphila* abundance was significantly associated with lower serum triglyceride (TG) levels, glucose, and insulin regulation. Likewise, it was negatively related to the expression of genes involved in lipid metabolisms, such as *Scd1*, *Srebf1*, and *Ppara*.

Nakano and colleagues (2020) demonstrated the role of bilberry anthocyanins (BAs) in preventing the development of non-alcoholic fatty liver disease (NAFLD) induced by a 3-month intake of a Western diet (WD) in mice [37]. The study targeted hepatic markers associated with lipid accumulation, inflammation, and oxidative status, as well as their link with changes in the gut microbiota. Bilberry anthocyanins lowered the serum levels of liver damage indices, including aspartate aminotransferase (AST) and alanine aminotransferase (ALT) in both the normal diet (ND) and WD-fed mice. Interestingly, observations of host phenotypes correlated with an increased relative proportion of *A. muciniphila.* The WD-induced disrupted gut microbiome was attenuated when supplemented with anthocyanin-rich açai, increasing the relative abundance of *A. muciniphila*, *Bacteroides acidifaciens*, and *Parabacteroides.* Furthermore, John et al. (2019) [87] demonstrated the potential of Davidson’s plum, which is rich in anthocyanins, including cyanidin sambubioside, cyanidin glucoside, peonidin sambubioside, and peonidin glucoside, to ameliorate the symptoms of metabolic syndrome in a high-carbohydrate, high-fat diet (HFHD)-fed rat model. Supplementation of Davidson’s plum (8 mg of anthocyanin equivalent/kg/day) for eight weeks reduced plasma TG, visceral and abdominal fat mass, and inflammatory markers. Davidson’s plum supplementation increased the abundance of *A. muciniphila* in rats fed the obesogenic and corn starch diets but was significantly higher in the latter group. Whereas in this study, we did not analyze the mucus layer, increased mucus secretion can be induced by the additive action of fibers and anthocyanins, providing a better ecological niche for *A. muciniphila.* Many anthocyanin compounds have been demonstrated to improve mucosa condition and injury and to hamper inflammatory opportunistic microbes [89,90].

In another study, Pan et al. reported an increase in relative levels of *Akkermansia* spp., along with *Anaerostipes* and *Ruminococcus* spp. and a decrease in *Acetivibrio* spp. abundance in F-344 rats in response to the dietary administration of anthocyanin-rich black raspberry (BRB) powder for six weeks [88]. This work sustained the modulation of inflammatory biomarkers by the whole BRBs, the BRB-derived anthocyanins, or the BRB-derived residue fraction. The metabolism of phenolics by the gut microbiota was not analyzed in that study; however, the authors indicated the potential of berry anthocyanins and fiber-bound phenolics to reduce gut inflammation, which is a possible indirect mechanism concomitantly sustaining a potential beneficial gut microbiota composition. It is worth noting that species of *Anaerostipes* spp., which were also increased by BRB, have the potential to degrade phenolic acids and are known producers of beneficial short-chain fatty acids such as butyrate and propionate. Notably, this genus harbors a functional gallate decarboxylase capability required to degrade gallic acid, as detected in silico and evaluated in vitro [91]. Interestingly, syntrophic interactions have been demonstrated between butyrate-producing species, such as *Anaerostipes caccae* and *A. muciniphila,* in a mucin-enriched medium [92]. In this sense, the butyrate producer benefits from the mucolytic activity of *A. muciniphila*, and in turn, the produced butyrate fuels the colonocytes and the goblet cells to support mucus production. Comparable complex cross-feeding chains might occur between polyphenol-degrader genera and *A. muciniphila*, although this hypothesis warrants further investigation. Subsequent in vitro mechanistic studies on the degradation of anthocyanins by *A. muciniphila* and identified polyphenol degraders are needed to translate the actual impact of the consumption of anthocyanin-rich foods on the stimulation of *A. muciniphila* and the enhancement of human metabolic diseases.

### 4.2. Flavan-3-Nol-Rich Foods

Flavanols are broadly characterized by the absence of a double bond between C-2 and C-3 and the absence of a carbonyl group on the C ring (C-4); instead, they have a hydroxyl group(s) on C-3 or C-4, where flavan-3-ols are the most reported [93] (Figure 2). Flavan-3-ol can contain a galloyllated and gallated group, including flavan-3-ol monomers, (+)-catechin, and (−)-epicatechin (EC), as well as oligomers, such as procyanidins, (−)-epigallocatechin (EGC)], (−)-epigallocatechin-3-gallate (EGCG), and (−)-epicatechin-3-gallate]. These compounds are widely distributed in foods such as cacao, teas, beans, red wine, apples, pomegranates, and berries [94].

The modulation of the gut microbiome by flavan-3-ols and the derived metabolites beneficially influences the metabolic host’s health. Dietary flavan-3-ols are widely associated with a cardioprotective and anti-inflammatory roles in coping with chronic diseases, including T2D, obesity, NAFLD, inflammatory bowel diseases (IBDs), and colon cancer [38]. Non-gallated flavan-3-ols, such as proanthocyanidins (PACs), also known as condensed tannins, are catabolized by the gut microbiota to phenylvaleric acid and valerolactones as primary metabolites [32,95]. Galloyllated flavan-3-ols, such as gallotannins and ellagitannins (ET), well-known as hydrolyzable tannins, are metabolized into ellagic acid and urolithin metabolites. Among the gut bacterial species known to metabolize flavan-3-ols are Lactobacilli, *Gordonibacter* spp., *Eggerthella lenta* (Eggerthellaceaea), and *Adlercreutzia equolifaciens* (Coriobacteriaceae) [44]. The derived flavan-3-ols metabolites are endowed with numerous biological properties, including antioxidant, antiatherosclerotic, antimicrobial, and anti-inflammatory activities [29,30,31,32].

The antiobesity effect of peach, a fruit rich in flavan-3-ols, was evaluated in female ICR mice fed an HFD [96]. The oral administration of peach peel extract (PPE), which is mainly composed of epicatechin 3-O-glucoside (28%) and proanthocyanins (12.7%), was shown to enhance intestinal and metabolic alterations. Unsurprisingly, the obesogenic diet resulted in a significant reduction in the relative proportion of *Akkermansia* spp., together with species of *Bacteroides, Bifidobacterium*, Lachnospiraceae_NK4A136_group, Prevotellaceae_UCG-001, *Alloprevotella*, and *Roseburia*. Moreover, it increased *Blautia*, *Bilophila*, *Odoribacter*, and *Ruminiclostridium* spp., among others. An intake of 300 mg/kg body weight (BW) of PPE and a lower concentration of 150 mg/kg BW significantly reversed the HFD-induced gut microbial changes after 12 weeks of intervention. Interestingly, the relative levels of *A. muciniphila* were recovered and even rose to levels higher than in the non-obese counterparts, doubling in abundance with the highest concentration of PPE of 300 mg/kg BW. These changes prevented obesity by reducing BW gain compared to the untreated HFD-fed mice, which is consistent with the reduced adipose tissue accumulation, decreased oxidative damage, and improved serum and liver lipid levels.

Accumulating studies point to proanthocyanins and ellagitannins as promising phenolic compounds to efficiently target the relative abundance of *A. muciniphila* [44] (Table 2 and Table 3). This might be related to the structural features of tannins, such as the degree of polymerization, the high proportion of hydroxyl and galloyllated groups, and their high affinity with membrane proteins [97,98,99]. Owing to these characteristics, tannins exhibit significant antimicrobial action against opportunistic pathogens found flourishing in metabolic and inflammatory diseases [98]. In previous work, we demonstrated that berry polyphenolic fractions mainly composed of anthocyanidins, as well as phenolic acids, oligomeric PACs, and polymers of PACs, distinctly modulated the gut microbiota of mice fed an obesogenic diet for eight weeks [100]. In this study, blueberry polyphenolic fractions were administered in equivalent proportions to those found in the whole extract. Remarkably, *A. muciniphila* was mainly stimulated by blueberry PAC-rich fractions but not by anthocyanins and phenolic acids in HFD-fed mice. 

In agreement with the selective effect of PACs on *Akkermansia*, Neto et al. (2021) [101] showed that a cranberry juice extract enriched with 57% PACs increased relative levels of *A. muciniphila* and intestinal mucus accumulation in mice with a simplified human microbiome (consisting of 25 predefined species) after 10 days of treatment. Interestingly, this expansion coincided with an increase in the relative abundance of *Bacteroides ovatus* and butyrate-producing taxa such as *Clostridium hiranonis*. In fact, cranberry juice supplementation did not increase the relative levels of *A. muciniphila* when it was inoculated alone in a gnotobiotic mouse model; in contrast, it increased the levels of *A. muciniphila* when it was in a microbial community. This observation suggests that *A. muciniphila* could proliferate at the expense of other microbes and PAC-induced mucin secretion. Various studies using blueberry and cranberry polyphenols, as well as fibers, were performed in obese mice, confirming that berry polyphenols but not fibers selectively promote the relative abundance of *A. muciniphila,* bearing health benefits [102]. This outcome was reproduced by oral supplementation of cranberry flavan-3-ols-rich extract administered separately but not with agave inulin in HFD-fed mice for eight weeks [103]. Indeed, the presence of inulin in the diet abolished the stimulating effects of polyphenols on the *A. muciniphila* levels, although this diet promoted other beneficial microbes. Interestingly, only the diet enriched with polyphenols but not inulin significantly increased the mucin-secreting goblet cells in obese mice [103].

Grape PACs have also been shown to increase the relative proportions of *A. muciniphila, Bacteroides* spp. and *S24-7* and to affect those of *Clostridium* spp., Lachnospiraceae, and Ruminococcaceae [104,105]. In addition, the increase in the relative *A. muciniphila* proportion by PACs might depend on the relative abundance of this bacterium at the baseline of the dietary treatment [106]. Masumoto et al. (2016) [107] showed a similar shift in relative levels of *A. muciniphila* by administering apple polymeric PACs (0.5%) to mice fed an HFHS diet for 20 weeks. The PAC-rich diet increased the relative proportions of *Akkermansia* spp., as well as *Adlercreutzia*, *Roseburia*, *S24-7*, and *Bacteroides* spp. taxa. In contrast, the proportion of *Clostridium*, *Lachnospiraceae*, and *Bifidobacterium* spp. was reduced. Here, the Firmicutes/Bacteroidota ratio was reduced to the level observed in the mice fed a standard diet. The PAC polymers significantly improved lipid metabolism and decreased the gene expression of hepatic lipopolysaccharide (LPS) receptors (*Tlr4* and *Cd14*), concurrent with reduced inflammation, enhanced intestinal permeability, and the inhibition of HFD-induced opportunistic microbes in the gut microbiota.

Camu-camu (*Myrciaria dubia*) is an Amazonian fruit rich in ET, galloylated PACs, and ellagic acid. The dietary supplementation of camu-camu extract (200 mg kg^−1^) in male HFHS-fed C57Bl/6J mice prevented obesity and improved glucose homeostasis. Gut microbiota analysis showed that flavan-3-ol-rich camu-camu decreased the abundance of *Lactobacillus* spp. and promoted *A. muciniphila, Barnesiella* spp. and *Turicibacter* spp. [108]. In agreement with this study, Abot and colleagues (2022) confirmed the promoting effects of camu-camu polyphenolic extract on *A. muciniphila*, even when administrated at a lower dose of 62.5 mg/kg to mice with HFD-induced obesity [109].

The stimulation of *A. muciniphila* by flavan-3-ols has also been shown to play a role in the attenuation of liver-related diseases. For instance, Xia and colleagues (2021) [110] recently evaluated the potential of catechin-rich Zhenjiang aromatic vinegar extract to modify gut microbiota composition and liver injury induced by long-term alcohol consumption in ICR male mice. Notably, ethanol administration increased the Firmicutes/Bacteroidota ratio, which was decreased by administering the vinegar catechin-rich extract. At the genus level, the relative abundances of *Akkermansia*, Lachnospiraceae_NK4A136_group, and *Bacteroides* spp. were significantly increased. These results demonstrate the role of polyphenols in stimulating *A. muciniphila* and reversing ethanol-induced gut disruption.

Polyphenol-rich teas are consumed worldwide and associated with health benefits. Teas mainly contain gallated flavan-3-ols, such as EGCG and ECG, as well as non-gallated flavan-3-ols, such as EC and EGC [115]. These compounds effectively prevent cardiovascular diseases. For instance, supplementing 2% green tea extract (GTE) containing EGCG (48%) was shown to impact the gut microbiota significantly, mainly by promoting *Akkermansia* and *Bifidobacterium* spp. in male C57BL/6J mice fed an HFD for eight weeks. At the phylum level, the GTE increased the relative abundance of Actinobacteria and Verrucomicrobia but reduced that of the Firmicutes. Species-level analysis indicated that *A. muciniphila, Bifidobacterium pseudolongum*, and *Bifidobacterium adolescentis* were significantly stimulated by the catechin-rich green extract compared to the HFD-fed untreated mice. It is worth noting that *Bifidobacterium* spp. are reported to be involved in flavan-3-ol metabolism and the release of valerolactones metabolites [116,117]. The derived metabolites exert additive protective effects against intestinal inflammation, improving the mucosa microenvironment status. GTE enhanced adipose inflammation and TJPs in the ileum and colon of HFD-induced obese mice, preventing metabolic disorders associated with obesity [112].

The modulation of the gut microbiota by different teas, including green, oolong, and black tea, was evaluated by Liu and colleagues in a mouse model of HFD-induced obesity for 13 weeks (Table 3) [113]. Although the specific proportion of phenolic classes was not detailed in the tea infusions, flavan-3-ols and flavonols were the major groups among the identified compounds. The green tea contained 3332.35 ± 70.91 mg L^−1^ total polyphenols, oolong tea contained 2911.52 ± 51.51 mg L^−1^, and black tea contained 2732.11 ± 23.64 mg L^−1^. All tea infusions recovered the microbial diversity and increased relative levels of *Akkermansia* spp., together with that of *Alistipes* spp., Lachnospiraceae, and *Rikenella microfusus*, and decreased those of *Allobaculum* spp., *Bacteroides acidifaciens*, *Clostridium leptum*, and *Parabacteroides goldsteinii* compared to HFD. The tea infusions prevented BW gain and fat tissue accumulation and decreased serum glucose, lipid, and lipopolysaccharide-binding protein (LBP) to levels comparable to the group fed a low-fat diet [113]. In the study, the relative increase in *Akkermansia* associated with tea-based drinks was significantly and negatively correlated with sserum LBP levels.

In concordance with the above findings, the daily administration of 0.32% EGCG to male HFD-fed C57BL/6N mice for eight weeks increased the abundance of the phyla Verrucomicrobia and Actinobacteria and the genera *Adlercreutzia* and *Akkermansia* but decreased the abundance of phyla Deferribacteres, Proteobacteria, and Firmicutes, as well as an unclassified genus of Desulfovibrionaceae, compared to the untreated HFD group [118]. The EGCG treatment inhibited BW gain, hepatic lesions size, and TG content in the liver of HFD-induced obese mice. Accordingly, a recent study evaluated whether the relative increase in *Akkermansia* was attributed to the EGCG compound from a green tea extract compared to the whole flavan-3-ol-rich extract. Again, the increased *Akkermansia* proportion was reproduced by an equivalent concentration of EGCG in obese and lean mice [119].

Most of the above studies show, as a common denominator, the stimulation of the relative proportion of *A. muciniphila* concomitantly with that of polyphenol-degrader taxa, such as *Adlercreutzia* and *Bifidobacterium* (Table 2 and Table 3) [120,121,122]. This observation suggests that *A. muciniphila* might benefit from flavan-3-ols degradation byproducts through the action of other microbes and the impact these compounds exert on its mucosal ecological niche. In another mechanistic study, it was demonstrated that polyphenols directly interact with intestinal mucin and reinforce the mucus layer. Feng et al. (2022) [123] evaluated the effects of EC, EGCG, and tannic acid (TA) on the intestinal mucin and barrier function through isothermal titration calorimetry and multiple-particle tracking using ex vivo mucus and Caco-2/HT29-MTX cocultures. Strikingly, it was demonstrated that gallated polyphenols and TA strongly bind to intestinal mucin, reinforce the mucus layer, and counteract inflammation [123]. 

Because polyphenols can significantly increase the viscoelasticity properties of the mucin-rich mucus layer, the colonization of opportunistic pathogens and LPS penetration might be substantially limited, thus contributing to the amelioration of the gut microbiota and intestinal disruption. These results can explain the stimulation of *A. muciniphila* by ellagitannins, in addition to providing a selective landscape for this bacterium to thrive in vivo.

Recently, Xia et al. (2021) [54] assessed the in vitro growth of *A. muciniphila* in a culture medium enriched with EGCG alone as a unique carbon source and in a medium containing EGCG and either mucin or glucose. Whereas *A. muciniphila* could not grow in the EGCG-enriched medium, it was reported to grow better when both EGCG and mucin were present in the medium than with only mucin. The growth improvement of *A. muciniphila* was dose-dependent, showing a gradual increase proportional to increasing concentrations of 50, 350, and 500 mg L^−1^ of EGCG. However, it should be noted that in this study, the *A. muciniphila* growth curve was measured by optical density (OD600), which is not a suitable method when high concentrations of polyphenols are added into the media because the presence of increased polyphenol interactions with mucin and proteins in the growing media provoke the formation of clumps, resulting in increased OD when the concentration of polyphenols increases. In this work, phenolic products were also analyzed in the media with and without EGCG. The presence of catechin gallate, gallocatechin, epigallocatechin, gallic acid, esculetin, hydroxyhydroquinone, and 3,4-dihydroxybenzaldehyde was reported only in *Akkermansia*-inoculated EGCG-enriched media, suggesting that *A. muciniphila* partly degrades EGCG in a yet unknown pathway. 

Moreover, it is unclear whether the phenolic products described in the EGCG fermentation by *A. muciniphila* with mucin or glucose might also result from the spontaneous hydrolysis [124] of this compound in the non-inoculated media, as the metabolic output of the control medium is not detailed in this study. Although this is an interesting approach to examining the prebiotic effect of polyphenols on *A. muciniphila*, the microbial enzymes responsible for ellagitannin trophic utilization, such as tannases and gallate decarboxylase, remain to be identified and tested in *A. muciniphila.*

In 2017, Henning and colleagues [43] reported the participation of *A. muciniphila* only in the hydrolysis of pomegranate ellagitannins (ETs) (punicalagin and ellagic acid) but not in their complete catabolism pathways to further produce the final metabolite, urolithin A. It has been demonstrated that in the human intestine, ETs and ellagic acid follow a lactone ring cleavage, decarboxylation, and dihydroxylation to form various metabolites, including urolithins. The notion of ET utilization by *A. muciniphila* arose from evidence for the stimulatory effects of ET (1000 mg of pomegranate extract daily) on *A. muciniphila* in humans associated with a healthier metabolic status and better clinical outcomes. A compelling finding from this study was that the relative levels of *Akkermansia* spp. were significantly higher in stool samples of urolithin-A producers (so-called metabotype A) compared to non-producing subjects [52,53]. Consequently, Henning and colleagues aimed to confirm the in vitro degradation of these phenolics in monoculture with *A. muciniphila* [43]. The authors demonstrated that *A. muciniphila* growth was significantly inhibited by the pomegranate ET (0.18 mg/mL and 0.28 mg/mL) and slightly reduced when grown only with ellagic acid (50 µM). Notwithstanding, ellagic acid increased when *A. muciniphila* was grown in a medium enriched with pomegranate ET, suggesting ellagitannin hydrolysis by *A. muciniphila*. Similarly, ellagic acid levels decreased when *A. muciniphila* was grown with only ellagic acid. However, phenolic metabolites other than urolithin A could be produced, as this was not detected in the media. Collectively, these findings the premise that *A. muciniphila* might prime phenolic degradation by other microbes by initiating the hydrolysis of parent compounds. In turn, it takes advantage of the effects of the phenolic metabolites in the mucosal microenvironment and microbial counterparts.

### 4.3. Flavonol-Rich Foods

Flavonols have a plane 3-hydroxyflavone base distinguished by hydroxyl groups in benzene rings [125]. Quercetin, myricetin, and kaempferol are good representatives of this phenolic group. Quercetin is widely found in apples, teas, berries, and onions. The gut bacterial species of *Eubacterium ramulus*, *Escherichia coli, Streptococcus lutetiensis, L. acidophilus, Weissella confusa, Enterococcus gilvus, Clostridium perfringens*, *Bacteroides fragilis*, and *Bacillus subtilis* have been reported to utilize quercetin [126,127]. The quercetinases, including a family of cupin-type dioxygenases, flavonol 2,4-dioxygenase, and quercetin 2,3-dioxygenases, have been reported to be involved in this activity [128,129,130].

Quercetin is a polyphenol with anti-inflammatory and cardioprotective effects. For instance, the role of quercetin in improving hypercholesteremia was demonstrated in LDLR^−/−^ (LDL receptor-deficient) C57BL/6 mice fed an HFD [131]. Quercetin supplementation (100 μg per day) reduced BW gain, atherosclerotic lesions, inflammation, and oxidative stress. The gut microbiota composition was significantly changed, with an increase in the relative abundances of Actinobacteria and Bacteroidota and a reduction in Firmicutes. At the genus level, quercetin supplementation increased the relative levels of the *Akkermansia*, *Bacteroides*, *Parabacteroides*, and *Ruminococcus* genera [132]. Similarly, Etxeberria et al. (2015) [133] investigated whether quercetin administration could prevent gut microbiota disruption induced by an HFHS diet for 6 weeks. Quercetin impacted the gut microbiota at different taxonomic levels. On the one hand, the Firmicutes/Bacteroidota ratio and the growth of bacterial species associated with diet-induced obesity were decreased, including Erysipelotrichaceae, *Bacillus* spp. and *Eubacterium cylindroides*. In contrast, relative levels of *A. muciniphila* were shown to be increased by quercetin compared to the relative abundance detected in diet-induced overweight control rats.

The coadministration of quercetin with other phenolics has also been demonstrated to reverse obesity and gut microbial dislocation, in addition to stimulating *A. muciniphila* (Table 4). For instance, 30 mg kg^−1^ BW per day of quercetin jointly administrated with 15 mg kg^−1^ BW per day of resveratrol in HFD-fed Wistar rats for ten weeks improved obesity by modulating the gut microbiota. The combination of the two phenolics significantly increased the microbial diversity and the relative abundance of *A. muciniphila*, Ruminococcaceae_UCG-014, Bacteroidales_S24-7_group_norank, Ruminococcaceae_UCG-005, and the Christensenellaceae family in HFD-fed rats. Additionally, a reduction in *Lachnoclostridium* and *Bilophila* spp. in the quercetin and resveratrol group was observed compared to the HFD group. The rat obesity phenotype was enhanced by a reduction in BW, in addition to the attenuation of serum lipids and inflammatory markers [133]. Dietary supplementation with a *Smilax china* extract rich in quercetin and neochlorogenic acid to C57BL/6J mice for 12 weeks improved glucose tolerance and reduced BW, inflammation, and lipid metabolism. Within the gut microbiota, the relative abundance of Akkermansiaceae increased, and that of Desulfovibrionaceae, Lachnospiraceae, and Streptococcaceae decreased compared to the HFHS-fed group [134].

Quercetin was found to promote *A. muciniphila* either administered alone or combined with other phenolic compounds in rodents (Table 4). In previous work, we screened the presence of quercetinases in the *A. muciniphila* ATCC BAA-835 genome, finding two proteins with a conserved domain of quercetinases, notably a cupin domain-containing protein encoded by Amuc_0801 and γ-carboxymuconolactone decarboxylase encoded by Amuc_1806 [44]. However, the enzymatic activity involved in quercetin degradation remains to be established.

Recently, it was demonstrated that a synergic effect of *A. muciniphila* (2 × 10^8^ colony-forming units (CFU)/200 µL) conjointly administrated with quercetin (37.5 mg kg^−1^ day^−1^) could counteract obesity and NAFLD in an HFD-induced obese Wistar rat model [39]. After six weeks of HFD-induced obesity, the three-week dietary treatment with *A. muciniphila* in Wistar rats was associated with less body fat; however, coadministration of *A. muciniphila* with quercetin boosted steatosis remission and improved hepatic lipogenesis modulation. Significantly, *A. muciniphila* intestinal colonization was increased when coadministered with quercetin, suggesting that the gut microenvironment created by quercetin intake determined the ability of *A. muciniphila* to proliferate within the gut microbiota. In particular, *A. muciniphila* correlated with liver lipid and bile acid metabolisms, supporting the role of this bacterium in many comorbidities, including metabolic syndrome and hepatic damage.

Flavonols also occur in glycosylated form, bound to glucose, rhamnose, or rutinose. The most common glycosidic form of quercetin is rutin (a glycoside combining quercetin and the disaccharide rutinose). The α-L-rhamnosidases intervene in rutin utilization via gut microbial species; *Bifidobacterium catenulatum* and *B. pseudocatenulatum* were shown to degrade glycosylated flavanols [135]. Recently, Riva and colleagues [136] identified which gut microbes were metabolically active after the amendment of rutin using a fluorescence-based single-cell activity measure (biorthogonal non-canonical ammino-acid-tagging (BONCAT)) combined with fluorescence-activated cell sorting (FACS). Lachnospiraceae (*Lachnoclostridium* and *Eisenbergiella*), Enterobacteriaceae, Tannerellaceae, and Erysipelotrichaceae species were included in the rutin-responsive fraction of the gut microbiota. Specifically, Enterobacteriaceae was associated with the conversion of rutin to quercetin-3-glucoside (Q-glc), and Lachnospiraceae was associated with quercetin (Q) production [136]. Although Verrucomicrobiaceae was not included within the rutin-responsive group, this study uncovered the contribution of other bacterial taxa in degrading flavonols and how they might support *A. muciniphila* colonization in the gut.

Flavonol glycosides have also been shown to protect against intestinal barrier disruption, inflammatory responses, and colitis by regulating gut microbiota composition, especially by increasing *A. muciniphila.* Bu et al. (2021) [137] evaluated the effect of flavonol-rich extract of *Abelmoschus manihot* flowers containing quercetin-3-O-robinobioside, gossypetin-3-O-glucoside, quercetin-3′-O-glucoside, isoquercetin, hyperoside, myricetin, gossypetin, and quercetin in DSS-induced colitis in C57BL/6J mice. The flavonol glycoside-rich extract remarkably increased the relative proportion of *A. muciniphila*, along with *Gordonibacter,* a well-known polyphenol-degrader genus (Table 4). These changes were reflected in the attenuation of the colonic inflammatory response and intestinal epithelial barrier dysfunction. The role of *Akkermansia* in protecting against colitis was further confirmed by daily administration of *A. muciniphila* cells (3 × 10^8^) to DSS-induced mice, demonstrating that this bacterium can reduce inflammatory markers, restore intestinal barrier function, and relieve colitis. Overall, these studies highlight *A. muciniphila* as an intermediary player in the potentialization of the protective role of flavonols in treating intestinal disruption and gut inflammation.

### 4.4. Flavanone and Flavonone-Rich Foods

Flavanones, such as hesperidin, eriodictyol, and naringin, are the most studied compounds in lemons, oranges, and berries. Naringin and hesperidin reach the colon intact [19], where, through the action of gut microbial α-rhamnosidase and β-glucosidase, the aglycones hesperetin and naringenin are generated. Hesperetin and naringenin can be subsequently metabolized into various phenolics, including dihydrocaffeic acid, isoferulic acid, 4-hydroxyphenylacetic acid, dihydroferulic acid, ferulic acid, resorcinol, phloroglucinol, phloretic acid, phloroglucinol acid, hydrocinnamic acid, 3-(3′-hydroxyphenyl), propionic acid, protocatechuic acid, and hippuric acid [138]. Among gut microbial species, *B. catenulatum* and *B. pseudocatenultum* have been reported to have the ability to hydrolyze hesperidin [135]. These compounds have the therapeutic potential to modulate several cardiovascular disease (CVD) risk factors. For instance, glucose-lowering, obesity-preventing, lipid-regulating, antioxidant, and anti-inflammatory effects have been reported in diabetic and obese models. More information about the health benefits of flavanones and derived metabolites can also be found in a review by Mas-Capdevila (2020) [139]. Liu et al. (2020) [140] evaluated the effects of hesperidin (100 or 200 mg/kg BW) in HFD-fed male C57BL/6 mice for 10 weeks. *A. muciniphila* was reduced in HFD-fed mice, but unexpectedly, it failed to be changed by hesperidin; instead, the treatment enriched *Lactobacillus salivarius* and *Desulfovibrio* and decreased *Helicobacter ganmani, Helicobacter hepaticus, B. pseudolongum*, and *Mucispirillum schaedleri* in HFD-fed mice. Whereas the relative abundance of *A. muciniphila* was not significantly modulated by hesperidin, gut microbiota modulation was correlated with reduced BW and obesity-related disturbances.

Examples of flavonones include luteolin and apigenin; apples, olives, and artichokes are good sources of these compounds. Within the gut microbiota, *Eubacterium ramulus* has been shown to metabolize apigenin, as well as quercetin, naringenin, daidzein, and genistein. This is attributed to the action of a phloretin-hydrolase to break the phloretin C-C bond [130,141,142]. Apigenin can enhance the production of butyrate and propionate, which are associated with mucin production [143], protecting the gut lining and facilitating the growth of mucin-dependent gut bacteria such as *A. muciniphila.* The stimulation of *A. muciniphila* by apigenin supplementation in animal models supports the above notion. It was demonstrated that apigenin administration to male C57BL/6 J mice for 16 weeks could alleviate metabolic endotoxemia by improving intestinal disruption and restoring gut barrier function induced by an HFD [144]. Augmentation of *Akkermansia* at the genus level potentially mediated the protective effects of apigenin on metabolic syndrome. Indeed, the metabolic phenotype, including BW loss and changes in gut microbiota composition, could be transferred from apigenin-fed mice to HFD-feeding mice via fecal microbiota transplantation [144].

### 4.5. Phenolic Acid-Rich Foods

Phenolic acids are predominantly found bound to the fiber fractions of vegetables, fruits, and cereal. Berries, red cabbage, rice, and wheat bran are good sources of phenolic acids [145]. Free phenolic acids are also found in coffee and cacao. Chlorogenic acid and caffeic acid, the predominant phenolic acid in coffee, can modify the gut microbiota structure, reducing intestinal and systemic inflammation in colitis. Notably, in the colon, chlorogenic acid is hydrolyzed into caffeic acid metabolite by mucosal and microbial esterase in the intestinal tract [146,147]. Both chlorogenic and caffeic acids significantly increased the relative abundance of *A. muciniphila* and the microbial richness, in addition to reducing the ratio of Firmicutes to Bacteroidota in mice with DSS-induced colitis [148,149] (Table 5). For instance, the effects of dietary caffeic acid (1 mM, CaA) administered for 15 days on murine experimental colitis were shown to contribute to coping with DSS-induced intestinal inflammation and gut microbiota dislocations. CaA significantly suppressed inflammatory markers, such as the secretion of IL-6, TNFα, and IFNγ and the colonic infiltration of CD3+ T cells, CD177+ neutrophils, and F4/80+ macrophages. Furthermore, caffeic acid has been shown to hamper the abundance of inflammatory Ruminococcaceae species [149,150].

Released fiber-bound phenolic acids, after in vitro digestion and colonic fermentation, also showed a significant prebiotic effect on *A. muciniphila*, *Faecalibacterium prausnitzii*, *Bifidobacterium* spp. and *Lactobacillus* spp. in in vitro studies [151]. The bound phenolics, including p-coumaric acid, hydroxybenzoic acid, and ferulic acid, exhibited excellent antioxidant and hypoglycemic activities. Interestingly, the dietary fibers removed by polyphenols did not show the same promoting effects on the gut microbiota [151]. This observation highlights the phenolic compounds as an important factor supporting trophic interactions with other gut microbes, leading to *A. muciniphila* proliferation. The enzymes involved in phenolic acid metabolism, such as feruloyl esterases (also called hydroxycinnamoyl esterases) and phenolic acid reductases, have been reported in *Bifidobacterium* and *Lactobacillus* genera. In contrast, owing to its polysaccharide-degrading activity, *F. prausnitzii* intervenes in releasing fiber-bound phenolic acids. The ability to utilize phenolic acids has been reported in *B. longum*, *Lactobacillus helveticus*, *Lactobacillus johnsonni*, *Limosilactobacillus reuteri, L. acidophilus, Limosilactobacillus fermentum*, *Latilactobacillus curvatus, Furfurilactobacillus rossiae*, and *L. plantarum* [152,153,154]. However, phenolic acid reductase and feruloyl esterase activities remain to be assessed in *A. muciniphila*.

### 4.6. Stilbene-Rich Foods

Stilbenes are found in grapes, cranberries, and red fruits. Resveratrol is a stilbene compound with several therapeutic effects on health, including protective properties against atherosclerosis and metabolic syndromes [155]. The microbial degradation of resveratrol yields 3,4′-dihydroxy-trans-stilbene and 3,4′-dihydroxybibenzyl (lunularin) metabolites. 

Recently, lunularin was reported to be further dehydroxylated by the gut microbiota, although only at the 3 position, to yield 4-hydroxydibenzyl, a novel metabolite found in human urine after resveratrol intake in healthy individuals [156]. The microbial species *Slackia equolifaciens*, *Adlercreutzia equolifaciens*, *Eggerthella lenta* (ATCC 43055), and *Bacteroides uniformis* (ATCC 8492) have been identified as dihydroresveratrol producers [156,157]. Regarding the impact of resveratrol and its relationship with the microbiota, there is evidence of it stimulatory effects on *A. muciniphila* [51] (Table 6). Resveratrol attenuated trimethylamine-N-oxide (TMAO)-induced atherosclerosis in ApoE^−^/^−^mice, decreasing TMAO concentration by modulation of the gut microbiota. Primarily, resveratrol increased the relative abundance of *Akkermansia, Bacteroides*, *Lactobacillus*, and *Bifidobacterium* spp.; instead, the relative levels of *Prevotella*, Ruminococcaceae, *Anaerotruncus*, *Alistipes*, and Peptococcaceae were decreased [158].

Resveratrol attenuated gut microbiota and intestinal disruption and reduced metabolic endotoxemia and colon inflammation in HFD-fed rats for six weeks [159]. This coincided with an increase in the relative abundance of *A. muciniphila* and reduced bacterial invasion in the distal colon. Likewise, increased tight-junction proteins (TJPs) and decreased FAK, MyD88, and IRAK4 were observed. Furthermore, resveratrol inhibited HFD-induced cannabinoid receptor type 1 (CB1) mRNA and suppressed CB2 mRNA levels in the colon. These data indicate that the endocannabinoid system plays a crucial role in resveratrol-induced protection against non-alcoholic steatohepatitis by maintaining gut barrier integrity and inhibiting gut inflammation. Resveratrol was also shown to attenuate colonic inflammation and clinical symptoms in a murine model of 2,4,6-trinitrobenzenesulfonic acid (TNBS)-induced colitis [160]. It restored the gut microbiota to homeostatic levels and increased butyrate production. At the species level, the relative levels of *A. muciniphila* were significantly increased in the TNBS + resveratrol group compared to the TNBS group [160].

Supplementation with resveratrol and pterostilbene in combination with a strictly calorie-restricted diet drives a beneficial microbial profile characterized by a relative increase in the level of the *Akkermansia* [161,162]. The activity of pterostilbene, a stilbene and a dimethoxy resveratrol derivative, may differ from that of resveratrol, but both molecules have been reported to exhibit similar cellular effects and mimic calorie restriction at the molecular level [162,163].

### 4.7. Lignan-Rich Foods

Lignans, such as secoisolariciresinol diglucoside (SDG), are present in berries, legumes, cereals, and especially in flaxseed. Most of the biological activities of lignans depend upon colonic bacterial transformations. The removal of glucose moiety from SDG, which the first step of the change, is performed by strains producing β-glucosidases. Lignan-hydrolyzing taxa include *Lactobacillus* spp., *Bifidobacterium* spp., Bacteroidales spp. and Clostridiales spp. Other species, such as *E. faecalis, E. lenta, B. producta, Eubacterium limosum, Clostridium scindens,* and *Lactonifactor longoviformis*, have been shown to utilize lignans [164,165]. Aglycones are further transformed into the enterolignans enterodiol (ED) and enterolactone (EL), exerting improved bioavailability and biological activity. This is the case of phytoestrogens, which were shown to protect against a chemically induced breast cancer model [166]. Contrary to other phenolic categories, lignans have been shown to decrease relative levels of *Akkermansia* spp. (Table 6). For instance, flaxseed lignans decreased the relative levels of *A. muciniphila* by 30-fold, increasing those of *Prevotella* and *Roseburia* spp. by 20-fold and 10-fold, respectively, in mice fed a basal diet for three weeks [167]. Another recent study confirmed the reduction in *Akkermansia* spp. and the increase in *Lactobacillus* and *Bifidobacterium* spp. in association with feeding with a high dose (50 mg/kg BW) of lignan syringaresinol in middle-aged mice [168]. The mechanisms underlying this coexclusion relationship between lignan derivatives and *Akkermansia* require further investigation. 

## 5. Modulation of *A. muciniphila* by Polyphenol-Rich Foods in Human Intervention Trials

A substantial body of literature has shown the modulatory effects of polyphenols on the gut microbiome in animal models. Despite the limited number of clinical trials, consistent data on the gut microbiota composition and the stimulation of *A. muciniphila* have been observed. Accumulating studies show evidence of the effect of polyphenols on improving metabolic alterations in the context of cardiovascular diseases [169,170]. Again, most studies focusing on the bioavailability of phenolics and the metabolome of individuals consuming polyphenol-rich foods point to the gut microbiota composition as an essential dynamic to extract polyphenol benefits to counteract metabolic diseases. However, factors such as interindividual variations on the baseline gut microbiota composition, genotypes, race, sex, and uncontrolled dietary habits in humans make it challenging to elucidate the selective mechanisms of polyphenols in boosting *A. muciniphila* health benefits in the host.

The duration of the dietary intervention, the health conditions, the presence of inhabitant microbes with the ability to break down phenolics (so-called metabotypes), the dose, and the phenolic class are essential variables to consider when translating the outcomes from experimental animal studies to human clinical trials. For instance, different profiles of the metabolic fate of labelled polyphenols (i.e., glucuronidation, sulfation, and methylation) have been reported across species. EC metabolites differ between humans and rats and, to a somewhat lesser degree, between humans and mice, as demonstrated by the kinetics of the metabolism of the flavanol (−)-epicatechin [36]. Despite the concerns about the species-dependent metabolic equivalence between humans and rodents, the role of the gut microbiome in polyphenol catabolism remains the most relevant factor in both models. In this section, we review compelling human studies that have assessed the effects of polyphenols on host health, in addition to the gut microbiota composition and the identification of *A. muciniphila* (Table 7).

The effects of various polyphenols, either pure or in combination with other food compounds, on gut microbiota and metabolic phenotypes were studied in humans with cardiovascular diseases. For instance, the consumption of 80 g of oats rich in β-glucans (3.0 g) and polyphenol (56.8 mg) in mildly hypercholesterolemic subjects for 45 days significantly increased the relative abundance of *A. muciniphila* and *Roseburia* spp. [171]. Oat intake significantly reduced total cholesterol and LDL-C. In particular, *A. muciniphila, Roseburia, Bifidobacterium*, and *F. prausnitzii* correlated with oat-induced changes in plasma lipids and short-chain fatty acids (SCFAs). In another study, intake of a combination of EGCG and resveratrol (RES) (282 and 80 mg day^−1^, respectively) or placebo was evaluated in overweight and obese men and women for 12 weeks. EGCG capsules contained 94% EGCG (141 mg per capsule), and RES capsules contained 20% trans-resveratrol (40 mg per capsule). Analyses of taxon abundance by real-time qPCR assays showed that *A. muciniphila* was not significantly affected by the administration of EGCG+RES in men or women, alongside Firmicutes, Actinobacteria, Gammaproteobacteria, and sulfate-reducing and acetogenic bacteria. In this study, the relative abundance of Bacteroidota was higher in men than women; EGCG+RES decreased its abundance in the same group, which correlated with improved fat oxidation [172]. These findings highlight that the effect of polyphenols relies on the interindividual differences linked not only to sex but also to the baseline of the microbiota composition. However, because qPCR assays target the changes of only specific taxa, a complete analysis of the whole gut microbiota is required to better understand the effects of polyphenol consumption on the abundance and function of other microbes.

In another study, Walker and colleagues [161] conducted a double-blind, placebo-controlled clinical trial and further assessed whether the impact of polyphenols on the gut microbiota was race-dependent (Table 7). The modulatory effects of polyphenols in the gut microbiota, especially the stimulation of relative levels of *A. muciniphila* and its impact on glucose metabolism and inflammation, were studied in obese men with metabolic syndrome [161]. The authors showed that oral supplementation of 1000 mg of resveratrol twice daily for 30 days improved glucose homeostasis in Caucasians but not in non-Caucasian obese men. Only in Caucasians, significant increases in relative levels of *A. muciniphila* were observed, coupled with improved insulin sensitivity and glucose homeostasis. It is worth noting that at baseline, the two groups presented with variable gut microbiota; on the one hand, Caucasian subjects had a higher concentration of *Collinsella*, Clostridiaceae, and *Ruminococcus* spp. than non-Caucasians, whereas *Streptococcus* and *Lactobacillales* spp. were over-represented in non-Caucasians. However, owing to the variability in the plasma levels of dihydroresveratrol, the primary resveratrol metabolite, the racial differences observed in the metabolic changes and relative *Akkermansia* abundance were not sustained [161], indicating that additional mechanisms other than microbial metabolism of phenolics could support the changes in *A. muciniphila* gut colonization and the improvements in glucose homeostasis.

In humans, it is difficult to conduct in-depth studies of the effect of polyphenols on the status of the mucosal colonic epithelium as an indirect outcome substantiating an improved niche for *A. muniniphila* to grow. However, the analysis of inflammatory markers in serum and fecal samples, such as C-reactive protein, IL-1, IL-6, and LBP, might support the amelioration of microbial LPS dislocation and the function of the gut epithelium barrier. However, these parameters were not studied in the above work. Likewise, extended dietary intervention coupled with metabolome analysis might yield broader outcomes with respect to the gut microbiota function associated with interindividual phenotypes beyond the gut microbiota composition.

Supplementation of Magnolia berry *Schisandra Chinensis* (SCF), a fruit popular in East Asian traditional medicine and rich in flavonoids, was studied in obese women for 12 weeks. The women were administered 100 mL of juice containing 12 mg total phenolic compounds and 3.34 mg total flavonoids twice a day (Table 7). At the genus level, the SCF induced an improved relative abundance of *Akkermansia*, *Roseburia*, *Prevotella*, *Bifidobacterium*, and *Bacteroides* spp. compared to placebo groups, as demonstrated by denaturing gradient gel electrophoresis analysis (DGGE) of the gut microbiota. Among the anthropometric and blood parameters, the juice decreased fat mass, fasting blood glucose, TG, AST, and ALT [173]. Fidélix and colleagues demonstrated a relative increase in *Akkermansia* spp., along with *Lactobacillus* spp. and *Ruminococcus* spp., after intervention with a flavanone-rich orange juice (300 mL d−^1^) for 60 days in healthy female volunteers. In addition, an inverse correlation was detected between these bacteria and glycemia, insulin, HOMA-IR, TG, total cholesterol, and LDL-C [40].

In a recent trial, the effects of mango on the intestinal microbiota and inflammatory markers were evaluated in patients with IBD. Mango is a rich source of flavonoids, such as quercetin, kaempferol glycosides, gallic acid, and galloyl glycosides. Patients received a daily dose of 300–400 g of mango pulp for eight weeks. Changes in the gut microbiota composition were followed by qPCR analysis. Whereas *A. muciniphila* was one of the targeted taxa, it was not reported to be modulated in the participants’ gut microbiota at the end of the intervention (Table 7). Mango mainly increased the relative levels of *Lactobacillus,* particularly *L. plantarum*, *Lactobacillus lactis*, and *L. reuteri*. These results are consistent with the capacity of *Lactobacillus* species to metabolize gallotannins. Daily intake of mango was shown to increase butyrate and decrease the plasma levels of proinflammatory cytokines, such as IL-8, growth-regulated oncogene (GRO), and granulocyte-macrophage colony-stimulating factor (GMCSF) [174].

Specific metabotypes might reflect an imprinted gut microbiota connected to the attenuation and progress of metabolic disorders and intestinal disruption upon the intake of polyphenol-rich foods. This feature has been reported with respect to the interaction between gut microbiota, isoflavones, ellagitannins, and ellagic acid [175,176]. For instance, previous research has underlined the role of certain gut bacteria, such as *Clostridium* spp. and two genera belonging to the Coriobacteriaceae family, *Gordonibacter* and *Ellagibacter* spp., in the conversion process of ET and ellagic acid into urolithin metabolites [177]. Metabotypes not only depend on the presence of polyphenol degraders in the gut microbiota but on the health status of the host. For example, the equol nonproducer metabotype (from isoflavone metabolism) and the urolithin metabotype B (formation of urolithin B and usourolithin A from ellagitannins metabolism) are more abundant in overweight and obese individuals [178,179,180]. Likewise, intraindividual variations in metabolite production in terms of stability over time are relevant in deciphering health benefits in the host [181]. Indeed, urolithin metabotypes were shown to be stable in humans. However, those individuals with the urolithin-nonproducing metabotype can acquire the benefits observed in urolithin producers after regular intake of ellagitannins and urolithin A [42], which can be transferred from breastfeeding mothers to their babies [182]. Notably, the latter observations indicate that dietary strategies rich in polyphenols may induce metabolic adaptions in the gut microbiota and re-establish the gut polyphenol-degrading enzymatic functions. In this context, Singh et al. (2022) showed that direct supplementation of urolithin A (500 mg) in healthy subjects with non-urolithin-producing features could provide the biological activities observed in urolithin A producers. Non-producers lacked the richness and diversity needed to transform the polyphenolic dietary precursors into urolithin A and had a lower Firmicutes-to-Bacteroidota ratio than high producers [42]. Furthermore, an increased abundance of *A. muciniphilia* was reported in the high urolithin A producers compared to the low and non-producers. This outcome was previously reported with a 47-fold higher proportion of this bacterium in urolithin A producers after a 4-week dietary intervention with pomegranate in healthy volunteers [52]. Analysis of *A. muciniphila* abundance was not tackled in subjects after the direct intake of urolithin A, given the short intervention with administering urolithin A mainly, with a focus on analyzing urolithin circulating levels after 24 h [42]. Here, urolithin A supplementation increased by sixfold relative to urolithin A glucuronide when compared with subjects receiving the pomegranate juice.

It is unclear whether the stimulation of this bacterium might stem from the intermediary byproducts generated by the initial hydrolysis of ellagitannins into ellagic acid and further urolithin metabolites. Trophic networking with other urolithin-producer microbes and the triggered homeostatic gut microenvironment might prevail more than the levels of urolithin A itself in the prebiotic effects of ellagitannins on *A. muciniphila.* Indeed, both ellagic acid and urolithin A could not directly prompt the growth of *A. muciniphila* in vitro [43]. The stimulation of this bacterium by the direct administration of urolithin A is yet to be confirmed in humans, although it has been demonstrated in animal models. Al Khalaf et al. (2021) [114] showed that oral supplementation of urolithin A and urolithin B (2.5 mg/kg each) induced the growth of *Akkermansia* spp. in rats fed a normal diet, with a higher proportion in urolithin-A-fed animals. In addition, only urolithin A significantly impacted the abundance of Firmicutes and Bacteroidota phyla. These latter findings might be associated with the antimicrobial potential of urolithin A as reported elsewhere [29]. It is also possible that inhibiting microbial competitors by polyphenol-derived metabolites might give *A. mucinihipla* an advantage in proliferating in the host gut.

The results of the above studies, including those performed in animal models, support the use of polyphenol-rich foods as therapeutic and dietary strategies to cope with gut inflammation, as well as liver and metabolic disturbances, boosting *A. muciniphila* as a critical intermediary.

## 6. Indirect Mechanism Favoring *A. muciniphila*: Polyphenol Signaling of the Host Gut Epithelium and Influencing the Gut Microbial Environment

The in vivo promoting effect of polyphenols on *A. muciniphila* embraces different routes that can directly or indirectly can enhance the colonization of this bacterium under steady and unsteady health conditions. It has been demonstrated that an HFD considerably perturbs the microbial composition, diversity, and functions of the gut microbiota and prompts mucosal inflammation and disturbed intestinal permeability. This is often associated with lower levels of *A. muciniphila* and a higher ratio of the major phyla Firmicutes and Bacteroidota in obese compared with lean subjects [183]. In this section, we tackle the impact of polyphenols on the restoration of the mucin-rich mucus layer and intestinal barrier function, as well as the role of polyphenols, such as antimicrobials, in reducing proinflammatory opportunistic microbes. These two factors provide *A. muciniphila* with a better gut microenvironment to proliferate and orchestrate regulatory anti-inflammatory and metabolic responses in the host.

### 6.1. Goblet Cells and Mucin Differentiation

Because *A. muciniphila* directly benefits from the increase in Muc2 secretion and improved mucus barrier [60,184], mucin modulation by polyphenols represents an indirect mechanism that prompts this bacterium. Polyphenols and microbial phenolic metabolites can actively signal the host epithelium, regulating mucin secretion and structure. A recent study mechanistically demonstrated that ellagitannins, ellagic acids, and urolithin metabolite can remodel the O-glycans profile by regulating the activities of N-acetyl-α-galactosaminyltransferases (ppGalNAc-T), preventing colorectal cancer cell migration and invasion [185]. Altered expression of mucin glycosylation has been documented in a variety of inflammatory, malignant, and metabolic diseases [23]. Crohn’s disease, ulcerative colitis, polyps, and colon cancer are associated with qualitative and quantitative changes in secreted sialomucins and sulfomucins and a consequently disrupted gut microbiota. Supplementation with polyphenols enhanced the mucosal barrier function by regulating the proportion of sialomucin mucin in rodents with 1,2-dimethylhydrazine-induced colon carcinogenesis [45]. Significantly increased sulfated mucin has been correlated with enhanced protection against enteritis [186]. In line with this, dietary polyphenols might be promising targets for therapeutic strategies by interfering with the function of specific glycosylation processes and glycosylated receptors.

Polyphenols have also been shown to modulate mucin biosynthesis without the action of the resident gut microbiota in a germ-free mouse model. Forgie et al. (2022) [27] demonstrated that diets rich pea seed coat proanthocyanins and hydrolyzable red-osier dogwood (ROD) tannins increased the luminal and fecal mucin GalNAc content in both germ-free and conventional Swiss–Webster mice after fourteen days of intervention. In particular, in mice harboring a conventional gut microbiota, the ROD extract increased *A. muciniphila* and *Bacteroides thetaiotaomicron*, along with an unclassified member of the Muribaculaceae family, and reduced *Romboutsia,* Ruminococcaceae, and Oscillospiraceae members.

The reduced relative abundance of *A. muciniphila* in obesity might correlate with a significant increase in the ratio of sialo/sulfomucins. Intake of an HFD suppresses GC differentiation and increases ER stress; thus, non-glycosylated Muc2 precursors accumulate and defect the mucin glycosylation process [24]. These alterations could influence the bacterial composition by decreasing the *A. muciniphila* abundance and gut microbial diversity. In this sense, polyphenols have been demonstrated to attenuate the disrupted glycan-rich ecological niche by increasing the mucus-secreting GC and reducing iNOS expression on the surface and in crypts of the colonic epithelium [26]. The mucosa restored by dietary polyphenols counteracted gut inflammation and recovered the relative proportion of *A. muciniphila* in HFD-fed mice. Likewise, Wang et al. (2020) [51] demonstrated that 16-week dietary administration of resveratrol upregulated TJP expression and mucins in mouse colon. Polyphenols might function as signaling molecules, contributing to GC maturation, the development of the intestinal epithelium, and the foundation of a healthy gut microbiota, especially *A. muciniphila*.

Recently, Lu et al. (2021) [187] showed that early-life dietary supplementation with grape polyphenols for 2 weeks increased the amount of mucus and Muc2 and led to a significant bloom of *A. muciniphila* population in post-weaning mice under specific pathogen-free (SPF) conditions. Notably, GC number and expression of the Kruppel-like factor 4, a marker of GC differentiation, and Cdx2, a Muc2-related promoter, was increased by dietary polyphenols after two weeks of intervention. This period was long enough to both promote an enrich the mucous environment for *A. muciniphila* and increase the population of *Lactobacillus* spp. The latter was shown to be an essential player in early-life colonization of the intestine, contributing to a boost in lactate levels and, in this case, the generation of phenolic metabolites due to its polyphenol-degrading enzymatic abilities.

### 6.2. Aryl Hydrocarbon Receptor (AhR) and Gut Inflammation

The aryl hydrocarbon receptor (*AhR*) is a ligand-activated transcription factor that regulates physiological processes in health and disease. Emerging studies point to the relevance of this receptor in binding to food and microbial agonists, resulting in beneficial outcomes in the host. The activation of this multifunctional sensor has been associated with protective roles in modulating energy and lipid metabolism, cell differentiation, and the immune system [188,189,190,191,192]. *AhR* agonists have been found to be decreased in sera of humans and animals suffering from alcoholic liver inflammation [193,194], multiple sclerosis [195], and metabolic syndromes, including obesity and diabetes [196]. This altered *AhR* regulation considerably influences the host gut microbiota, particularly *A. muciniphila* abundance. For instance, *A. muciniphila* and the *AhR* are depleted in the liver and colon of mice with saccharin/sucralose-induced NAFLD [197]. Reduced *AhR* agonists in mice sera were correlated with a disrupted gut epithelium and an increase in inflammatory markers [197].

Phenolic molecules have been reported as the main source of *AhR* modulators found in the human diet [48]. Isoflavones, quercetin, urolithin A, luteolin, and baicalein have been shown to act as the *AhR* agonists or antagonists [198,199,200,201]. A recent study evaluated the roles of polyphenol–microbe interplay in *AhR*-mediated signaling using a simulator of the human intestinal microbial ecosystem (SHIME) coupled with HepG2 and Caco-2 cell assays [199]. Microbiota-derived metabolites of tryptophan and flavonoids were assessed. Koper et al. (2019) first demonstrated a structure-dependent *AhR* activation by flavonoids in HepG2 and Caco-2 cells. Interestingly, a planar structure and the number of hydroxyl groups of polyphenols were found to be essential features modulating *AhR* activation [199]. In this study, luteolin and baicalein were identified as compounds activating the most *AhR* signaling. In addition, the luteolin-fermented metabolites derived from the ascending (AC), transverse (TC), and descending (DC) colon of the SHIME system were shown to drive the *AhR* activation differently. Notably, AC metabolites led to *AhR* activation, whereas a greater response was induced by the TC- and DC-derived metabolites, suggesting that in addition to dietary substrates, the gut microbiota is an essential supplier of endogenous *AhR* agonists, such as tryptophan derivates.

Tryptophan catabolism by the resident gut microbiota has also been shown to be influenced by dietary polyphenols, yielding physiologically relevant *AhR* ligands in the host. For instance, Marques and colleagues [201] showed that the administration of blackberry anthocyanin-rich extracts (25 mg kg^−1^ BW per day) changes the gut microbiota composition and tryptophan metabolism, increasing the production of the neuroprotective metabolite kynurenic acid. Indeed, adding polyphenols, together with tryptophan derivatives, significantly boosted the *AhR* response compared to the tryptophan derivatives alone in both rat DR-H4IIE and human DR-HepG2 hepatoma cells. Isoflavones (e.g., daidzein and genistein) and resveratrol the *AhR* response in DR-H4IIE cells increased by up to fourfold compared to tryptophan-derived ligands alone, such as TCDD (2,3,7,8-tetrachlorodibenzo-p-dioxin) and FICZ (6-formylindolo [3,2-b]carbazole). Furthermore, quercetin and both flavones baicalin and chrysin were shown to act as *AhR* agonists in human DR-HepG2 cells, regardless of the presence of tryptophan derivatives in the medium.

The role of the polyphenol–*AhR* interactions has been further demonstrated in *AhR*−/− mouse models using urolithin A, a metabolite of microbial degradation of ellagitannins. This metabolite was implicated in protection against gut inflammation in experimental colitis via the *AhR–Nrf2* pathway [200]. The activation of *AhR* by polyphenols and microbial metabolites results in the downregulation of inflammatory cytokines and the upregulation of *IL-22* and TJPs [47,199]. In this sense, the *AhR/IL-22* axis has been shown to be crucial in maintaining mucosal barrier integrity and in protecting the gastrointestinal epithelium against pathogen colonization [49,202,203,204,205]. Mechanistic studies have also demonstrated that reduced secretion of *IL-22* was correlated with impaired glycocalyx- O-glycan in mice suffering from obesity [206] and that such glycosylation pattern could be restored by boosting the *IL-22* levels [24,207]. The immunomodulatory outcomes linked to the *AhR/IL-22* axis contribute to changes in the gut microbiome composition, stimulating beneficial species such as *A. muciniphila* and reducing the susceptibility to metabolic and inflammatory gastrointestinal disorders.

**Figure 4:**
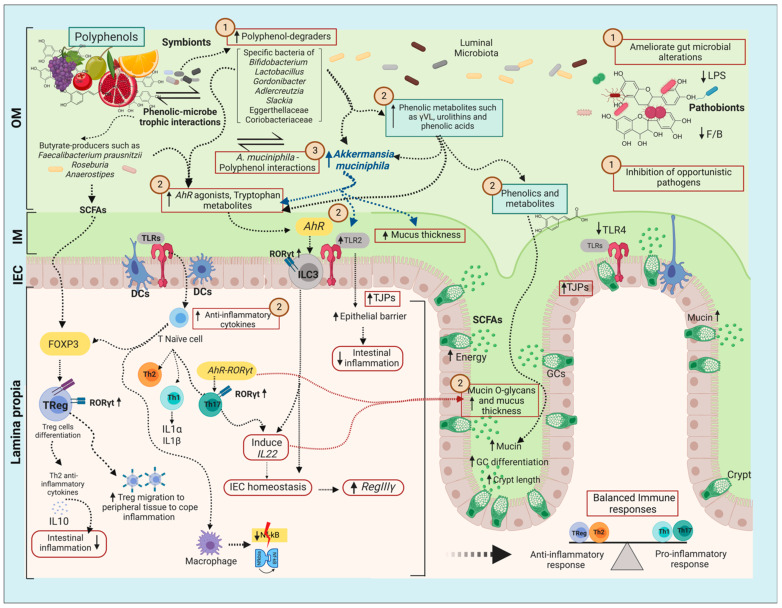
Pleiotropic polyphenol–microbiota interactions promoting an increase in *Akkermansia muciniphila* in the host gut. Metabolic diseases, such as obesity and type 2 diabetes, are associated with an altered intestinal ecological niche primarily associated with the onset of gut microbiota dysfunctions and a decrease in relative levels of *A. muciniphila*. The consumption of polyphenols, either pure or combined with other dietary susbstrates (e.g., fibers) shapes the gut microbiota composition, selectively promoting the relative abundance of *A. muciniphila* in association with improved metabolic outcomes in the host. Three main routes underline the stimulatory effects of polyphenols on *A. muciniphila* (represented by enumerate circles): (**1**) Interactions between polyphenols and the gut microbiota result in the inhibition of opportunistic pathogens, in addition to favoring symbiotics and the polyphenol-degrader microbes. Polyphenols reduce the level of lipopolysaccharides (LPS) and suppress the Toll-like receptor (TLR)-4, attenuating metabolic endotoxemia. Polyphenol-microbe trophic interactions yield phenolic metabolites, such as phenyl-γ-valerolactones (γVL), urolithins, and phenolic acids. De novo generated phenolics and the microbial metabolites trigger immunological pathways, restoring the gut epithelial function and homeostasis and positively affecting the abundance of *A. muciniphila*. (**2**) Notably, phenolics and tryptophan metabolites signal the Aryl hydrocarbon receptor (*AhR*). This activation controls cell recruitment, Th2 and Th17/IL-22 responses, and the remodeling of mucin O-glycans and intestinal epithelial cells (IEC) in an *IL-22* dependent manner. *AhR* activation in lymphocytes also influences cells recruitment and *IL-22* expression and improves intestinal inflammation. Then, the polyphenol-induced *AhR/IL-22* pathway provides *A. muciniphila* with a restored microecological niche. Polyphenols and *A. muciniphila* jointly act as activators of *AhR*, playing regulatory roles in intestinal cell function. *A. muciniphila* colonization contributes to a reduction in proinflammatory markers via TLR2 induction, helping to cope with low-grade local inflammation and promote epithelial tight-junction proteins (TJPs). In addition, SCFAs, such as butyrate, can drive the activation of the transcriptional regulator Forkhead box P3 (Foxp3), which is also linked to the upregulation of the anti-inflammatory IL-10. (**3**) *A. muciniphila* can directly be affected by phenolics and derivatives, influencing the expression of its molecular and metabolic attributes; it can hydrolyze phenolics and liberate smaller molecules that are substrates for other microbial analogs to subsequently generate bioactive phenolic metabolites. Overall, polyphenols favor anti-inframammary reactions, thus re-establishing the equilibrium between Treg/Th2- and Th1/Th17-associated immune response. The IEC can sense and absorb phenolic metabolites with health effects in the gut barrier’s permeability and distal organs and tissues. A hypothesis we cannot rule out is that some phenolics cause intestinal damage, resulting in increased mucus production and inducing *A. muciniphila* and improved barrier function as a kind of overshoot. *A. muciniphila* creates a positive feedback loop, thus renewing the mucus layer and maintaining its thickness. F/B: Firmicutes-to-Bacteroidota ratio; RAR: related orphan receptor gamma Rorγt; ILC3: innate lymphoid cell type; DC; dendritic cells; IM—inner mucus layer; OM: outer mucus layer. The graph was created with BioRender.

Bidirectional *AhR*-related effects might explain the *Akkermansia*-driven benefits in the host upon consumption of dietary polyphenols. On the one hand, polyphenols and derived microbial metabolites might activate *AhR*-dependent pathways, which restore the ecological niche of *A. muciniphila* (i.e., through IL-22, TJP regulation, and promotion of GC differentiation) [188,189,203,208]. On the other hand, phenolic compounds might potentiate the gut traits of *Akkermansia*, allowing this bacterium to orchestrate immunomodulatory markers and contribute to the maintenance of intestinal homeostasis. *A. muciniphila* and its membrane protein, Amuc_1100, can significantly increase gut indole loads and, consequently, *AhR* activation [71]. It is worth mentioning that Amuc_1100 is a well-known TLR2-inducer and substantiates the role of *A. muciniphila* in attenuating gut inflammation and metabolic disorders [14,17]. The mechanisms of action by polyphenols in sustaining an enhanced gut microecological niche promoting *A. muciniphila* colonization are illustrated in Figure 4.

### 6.3. Inhibitory Action on Gut Microbial Competitors

Unabsorbed dietary polyphenols and their metabolites can behave as activators or inhibitors of bacterial growth, depending on their chemical structure and concentration [209]. Phenolic metabolites and parent compounds selectively inhibit pathogens and stimulate the growth of commensal bacteria [44,143]. It has also been indicated that the modulatory effect of polyphenols on gut bacteria relies on the alteration of the cell physiology [210] rather than the reduction in the bacterial quantity per se. The expression of stress response and protein chaperone genes is upregulated in gut bacteria exposed to polyphenols, indicating the adverse, species-dependent effects of polyphenols on the gut microbiota [211]. The inhibitory mechanisms of action of phenolics have been compared with those exhibited by antibiotics [99]. For instance, based on the inhibitory effects and intracellular metabolites induced by apigenin against pathogens, this phenolic was clustered together with rifampicin and norfloxacin [212]. These antibiotics target RNA polymerase, gyrase, and topoisomerase IV, and apigenin could affect nucleic acid processing enzymes. Polyphenols can affect cell membrane/wall synthesis, for example, by altering the D-alanine: D-alanine ligase and the type II fatty acid synthetic pathway [213,214]; therefore, most of the Gram-positive gut bacteria have been shown to be more susceptible to polyphenol–antimicrobial actions; this is the case of the Firmicutes phylum, as expounded above in the section discussing the outcomes of animal studies. *A. muciniphila* has been stimulated by administering vancomycin and other wide-spectrum antibiotics [215,216]. In analogy to the antimicrobial actions of phenolic phenolics, reducing microbial competitors and associated inflammation allows *Akkermansia* spp. to flourish in the gut. In addition, in vitro studies of *A. muciniphila* growth in media enriched with polyphenols validate the resistance and adaptation of this bacteria to be metabolically active and resilient upon polyphenol intake. The antimicrobial effects of dietary polyphenols on food and gut microbial pathobionts associated with metabolic and inflammatory diseases were recently reviewed by Makarewicz et al. (2021) [28].

## 7. Polyphenol Effects on *A. muciniphila* Fitness: Hints of Metabolism and Adaptation to Polyphenol-Rich Foods

The physiological properties of *A. muciniphila* and the potential to adapt to polyphenols might be closely related to its genetic composition [57]. It harbors genes not only encoding mucin-degrading enzymes, accounting for 14% of the genome, but also genes linked to capsular and slime polysaccharide production. These features might support the tolerance of *A. muciniphila* to the cell wall/membrane disturbances provoked by polyphenols in gut bacteria. Proteomics and transcriptomics revealed that *A. muciniphila* molecularly adapts when exposed to disadvantageous conditions, such as a medium rich in bile acids, or when its favorite nutritional substrate, mucin, is depleted [58,217]. Notably, markers linked to acid and oxidative stress responses, nucleotide excision repair, inorganic ion transport and motility, ABC and RND (Resistance-Nodulation-cell division) transporters, and polysaccharide biosynthesis systems are upregulated in *A. muciniphila*. Additionally, changes in the metabolic profile of *A. muciniphila* are reliant on the substrate input, which determines its versatility and resilience to adapt in a less favorable medium [58], such as that induced by polyphenols. Intriguingly, mucin-depleted conditions have been shown to trigger attributes in *A. muciniphila,* supporting antiobesity effects in mice [218]. Polyphenols have been shown to modulate similar molecular functions of adaptation in bacteria with and without polyphenol-degrading potential, such as *Salmonella enterica, Escherichia coli, L. plantarum, Enterococcus caccae, Ruminococcus gauvreauii,* and *B. catenulatum* [143,211,219,220,221,222,223,224]. Under polyphenols-induced pressure, *A. muciniphila* might acclimatize to phenolic antimicrobial actions by altering its metabolic pathways and membrane phenotypes. The expansion of the relative *A. muciniphila* proportion in the gut microbiota upon polyphenols intake might be encouraged by polyphenol degradation by other microbes rather than the direct polyphenol utilization by this bacterium. As discussed above, many studies have shown an increase in *A. muciniphila*, along with other well-known polyphenol-degrader and polysaccharide-degrader microbes. This is the case of specific species belonging to *Lactobacillus, Bifidobacterium, Slackia, Gordonibacter, Eggerthella, Adlercreutzia,* and *Bacteroides* genera, which have been demonstrated to possess enzymes involved in the breakdown and utilization of different phenolic classes. Likewise, given their polysaccharide-degrading potential, species such as *F. prausnitzii* and members of *Roseburia*, *Prevotella*, and Clostridiales are concomitantly prompted with *A. muciniphila* by the intake of fiber-bound polyphenols encountered in several polyphenol-rich fruits and vegetables. Given the inferred trophic interactions that occur among polyphenol and polysaccharide degraders in vivo, both polysaccharide fermentation and subsequent polyphenol-degrading activity by other microbes may support *A. muciniphila* in the gut. A comparable case of crossfeeding upon polyphenol administration was reported between the polysaccharide degrader *Bacteroides thetaiotaomicron* and the polyphenol degrader *E. ramulus* [225]. Here, the release of glucose and maltose upon the fiber fermentation by *B. thetaiotaomicrom* drove the degradation of the flavonol quercetin by *E. ramulus*. These polyphenol-degrading and fiber-degrading activities reduced significantly when both species were grown separately. Similarly, in another study, *E. caccae* was shown to be effectively inhibited by apigenin when cultured alone, but this genus was enhanced when tested within a gut microbial community [143]. It still remains to be determined whether *A. muciniphila* utilizes the ensuing phenolic metabolites and the SCFAs formed from polyphenol-rich extract fermentation. Coculturing *A. muciniphila* with polyphenol-degrader microbes would provide insights into the notion that this bacterium is powered by intimate crossfeeding with other microbes upon polyphenol-rich food intake.

The direct trophic utilization of phenolics by *A. muciniphila* has not yet been investigated, and the identification of novel enzymatic activity responsible for polyphenol degradation remains to be mechanistically studied in vitro. Conflicting findings have been reported in a few studies dealing with the ability of *A. muciniphila* to utilize polyphenols in polyphenol-enriched media (i.e., EGCG, gallic acid, ellagitannins, and ellagic acid) [43,54]. On the one hand, *A. muciniphila* could not succeed in a medium enriched with EGCG as a unique nutritional source. In addition, aside from gallic acid, the specific phenolic end metabolites, such as phenyl-γ-valerolactones, phenylvaleric, and propionic acid derivatives, were not identified when it was grown optimally in an EGCG-enriched medium containing mucin [54]. On the other hand, *A. muciniphila* growth was shown to be hampered by pomegranate ellagitannins and ellagic acid. However, the levels of ellagic acid were shown to increase when this bacterium was grown with pomegranate ellagitannins [43]. The fact that both gallic and ellagic acids derived from the initial hydrolysis of galloylated and gallated parent flavan-3-ols were found in the growing media indicates that *A. muciniphila* primes the hydrolysis of EGCG and ellagitannins but does not participate in the subsequent phenolic degradation. However, this initial hydrolytic step might contribute to empowering the polyphenol-degrading action of other microbes, in turn furnishing the gut with bioactive metabolites and contributing to the maintenance of intestinal homeostasis.

Because the main nutritional source of *A. muciniphila* is mucins, its growth is not strictly confined to metabolizing dietary polyphenol-rich sources; however, the latter modulates the abundance and activity of other microbes and promotes mucin-secreting GC, giving *A. muciniphila* a colonizing advantage compared to its counterparts. The fermentation of different substrates, such as galactose, fucose, glucose, Glc-NAc, and Gal-NAc, by *A. muciniphila* has been shown to be enhanced when this bacterium is grown in the presence of mucin [58]. In analogy to the above observation, we suggest that polyphenols elicit distinctive metabolic and molecular phenotypes in *A. muciniphila*, in addition to increasing the mucin supply in the host. By degrading the newly polyphenol-induced mucins, *A. muciniphila* creates a positive feedback loop, renewing the mucus layer and maintaining its thickness. Considering all these studies, *A. muciniphila* proliferation stems from the synergic effects between crossfeeding networking with polyphenol degraders and the local impacts of phenolic derivatives on the mucin-rich mucus layer in vivo.

## 8. Conclusions and Future Directions

In the present work, we revisited preclinical and clinical trials demonstrating the prebiotic-like effect of polyphenolic extracts and polyphenol-rich foods on *A. muciniphila*. Different phenolic classes, including phenolic acids, anthocyanins, flavan-3-ols, flavonols, flavones, and stilbenes, distinctly stimulate *A. muciniphila* in the gut. For example, high intra- and interindividual variability in the gut microbiota composition impacts the fate of polyphenols and health benefits in the host and, consequently, the relative abundance of *A. muciniphila*. We highlighted three main routes underlying the polyphenol-induced *A. muciniphila* increase in the gut: (1) modulation of gut microbiota ecology while inhibiting microbial competitors and promoting the polyphenol-degrader consortium, (2) attenuation of gut inflammation and restoration of mucin-rich barrier function, and (3) direct trophic utilization and crossfeeding networking between *A. muciniphila* and other gut microbes. A conglomerate of bacterial taxa with polyphenol-degrading abilities often stands out concomitantly with an increase in *A. muciniphila* resulting from the consumption of polyphenol-rich foods in animals and humans. However, we cannot rule out that some phenolics signal the intestine, which results in increased mucus production, inducing the growth of *A. muciniphila* and improving barrier function, in addition to other metabolic benefits.

Few gut bacteria are known to degrade polyphenols and significantly correlate with *A. muciniphila* abundance. This is the case of ellagitannin degraders, yielding smaller molecules of urolithin A. Inconclusive results have been reported with respect to how ellagitannins and ellagic acid directly promote *A. muciniphila* in vitro, as reduced growth was observed when these compounds were added to the growing media. In a real-life scenario, it is most likely that the effects would be due to the trophic utilization of other microbes of ellagitannins and the immunomodulation that the urolithin A and parent compounds trigger in the gut.

It is yet to be determined whether *A. muciniphila* can metabolize different phenolic classes as single or combined substrates in monoculture and a community setting. Specific questions remain to be answered, such as which intermediary enzymes and degradation pathways are involved in polyphenol metabolism by *A. muciniphila*, as well as the resulting metabolic products. Moreover, there is a need to move from relative abundancies towards absolute abundancies and correct for the impact of transit time on the relative abundance of *A. muciniphila* and other intestinal bacteria [226,227]. A potential crossfeeding phenomenon behind polyphenol degradation might occur, as the metabolites from one bacterium could empower substrate utilization by another. A combination of omics tools, including transcriptomics, lipidomics, proteomics, and metabolomics, would provide a whole picture of the prebiotic effect of polyphenols on *A. muciniphila* that is more relevant than the structure of the gut microbiota itself. Polyphenols continue to receive a considerable amount of attention, owing to their selectivity to sponsor *A. muciniphila*, representing a cost-effective therapeutic and preventive approach with no demonstrated adverse side effects to cope with metabolic diseases.

Emerging coencapsulation techniques for the development of new synbiotics using polyphenols and probiotics are promising strategies for further application in nutritional therapies to counteract gut microbiota disturbances and metabolic syndromes [228,229,230]. Research has begun on the coencapsulation of *A. muciniphila* with polyphenols. Cells of *A. muciniphila* were successfully coencapsulated in succinate-grafted alginate doped with EGCG by spray drying [230]. Moreover, a recently marketed European product contains tablets of freeze-dried pasteurized *A. muciniphila* and other components, including ECGC (www.theakkermansiacompany.com (accessed on 31 October 2022)). In this sense, the inclusion of recognized polyphenol-degrading probiotics in the encapsulation preparations represents a promising avenue for the delivery of the goods of polyphenol–*Akkermansia* synergy to individuals with less favorable metabotypes.

## Figures and Tables

**Table 1:** Modulation of the gut microbiota by anthocyanin-rich foods in animal models.

Polyphenol-Rich Food		Experimental Design	Main Findings in the Gut Microbiota	*A. muciniphila* Modulation	Impact on Host Health	Ref.
**Anthocyanins**	Açai extract	SPF C57BL/6J mice fed a HFD for 14 weeks with daily gavage of 150 mg/kg of anthocyanin-rich açai extract	↑ *Parabacteroides distasonis* and *B. acidifaciens.*	↑ *A. muciniphila* was significantly and negatively associated with serum TG, glucose, and insulin	Enhanced liver damage, glucose intolerance, and insulin resistance	[86]
	Davidson’s plum	HCHF-induced obesity in Wistar rats supplemented with 8 mg of anthocyanins equivalent/kg/day for 8 weeks	↓ Clostridiaceae, ↑ *Turicibacter* spp.	↑ *A. muciniphila*	Reduction in visceral fat accumulation, inflammation, and plasma TG	[87]
	Bilberry	Western diet (WD)-induced NAFLD in male C57BL/6N mice for 18 weeks supplemented with 2% bilberry anthocyanins	↓ F/B, *Prevotella* spp., Lactobacillales, and Clostridiales; ↑*B. acidifaciens*; ↑*Parabacteroides* spp.	↑ *A. muciniphila* was negatively correlated with liver injury	Attenuated liver injury and dyslipidemia	[37]
	Black raspberries (BRBs)	5% whole BRB powder, 0.2% BRB anthocyanins or 2.25% of the residue fraction supplemented in F-344 rats under a standard diet for 6 weeks	5% whole BRB powder; ↑ *Anaerostipes*; ↑ *Ruminococcus*; ↑ *Coprobacillus*; ↓ *Acetivibrio*; ↓ *Anaerotruncus* spp.	↑ *A. muciniphila* by the whole BRB and the residue	Enhanced inflammatory biomarkers	[88]

The results relate to the relative abundance of taxa in fecal or cecal samples. BW, body weight; HFD, high-fat diet; HCHF, high-carbohydrate, high-fat diet; F/B, Firmicutes-to-Bacteroidota ratio; TG, triglycerides; non-alcoholic fatty liver disease (NAFLD).

**Table 2:** Impact of flavan-3-ol consumption on the relative colonic abundance of *A. muciniphila* and other bacteria.

Polyphenol-Rich Foods		Experimental Design	Main Findings in the Gut Microbiota	*A. muciniphila* Modulation	Impact on Host Health	Ref.
**Flavan-3-ols**	Wild blueberry and PACs fractions	HFHS-induced obesity in male C57BL/6N mice gavaged daily with 200 mg/kg BW of WBE or equivalent PACs for 8 weeks	↑2-fold Adlercreutzia equolifaciens* (Coriobacteriaceae) by the WBE and PACs	↑2.5-fold *A. muciniphila* (24.8%)	Enhanced glucose tolerance, ↑GC, restored colon mucus layer	[100]
	Cranberry juice (57% PACs)	Germ-free C57BL/6 with a simplified human microbiome gavaged daily with 200 mg/kg BW	↑*Bacteroides ovatus*, ↑*Clostridium hiranonis*	↑*A. muciniphila*	↑Intestinal mucus accumulation	[101]
	Cranberry (CP) and blueberry (BP) extracts (PACs) and their fibrous residues (CF and BF)	HFHS-induced obesity in male C57BL/6N mice fed 200 mg/kg berry powder or the equivalent fibrous fractions for 8 weeks	↓F/B, ↑*Dubosiella newyorkensis*, ↑*Angelakisella* spp., ↑Coriobacteriaceae* ↑Eggerthellaceae,*. ↓Lachnospiraceae, ↓Ruminococcaceae, ↓Peptostreptococcaceae	↑*A. muciniphila* by CP and BP correlated with lower BW	↓Fat mass depots, ↓BW, ↑mucus layer thickness	[102]
	Peach peel extract (PPE) (28% epicatechin 3-O-glucoside and 12.7% PACs)	HFD-induced obesity in female ICR mice fed 300 mg/kg (HPP) or 150 mg/kg (LPP) BW for 12 weeks	↓F/B. ↑*Lactobacillus* spp.,* ↑*Bifidobacterium* spp.,* ↑*Roseburia* spp., ↑*Bacteroides* spp.,* ↑Lachnospiraceae,* ↑Prevotellaceae, and ↑*Alloprevotella* spp.	↑*A. muciniphila* by HPP and LPP	↓BW, ↓oxidative stress, ↓hepatic lipid accumulation, ↑butyrate	[96]
	Cranberry extract (CP) (PACs) and agave inulin (AG)	HFHS-induced obesity in male C57BL/6N mice gavaged with either 200 mg/kg BW CP, 1 g/kg BW AG, or both for 9 weeks	↑*Muribaculum* spp., ↑*Faecalibaculum rodentium*, ↑*Roseburia* spp., ↑*Alistipes* spp. ↑Bacteroidaceae*, ↓*Ruminiclostridium* spp., ↓Lachnospiraceae, ↓Peptococcaceae	↑5.0-fold *A. muciniphila* only by CP	↑GC number, ↑Nlrp6, improved glucose tolerance, ↑TLR2, ↑AhR, ↓colon inflammation	[103]

* Some species belonging to this genus or family exhibit polyphenol-degrading abilities. BW, body weight; HFD, high-fat diet; HFHS, high-fat, high-sucrose diet; IP, intraperitoneal; F/B, Firmicutes-to-Bacteroidota ratio; NA, not applicable; TJPs, tight-junction proteins; GC, goblet cells; LBP, lipid and lipopolysaccharide-binding protein.

**Table 3:** Impact of flavan-3-ols and phenolic metabolites on the relative colonic abundance of *A. muciniphila* and other bacteria.

Polyphenol-Rich Foods		Experimental Design	Main Findings in the Gut Microbiota	*A. muciniphila* Modulation	Impact on Host Health	Ref.
**Flavan-3-ols**	Apple polymeric proanthocyanins (PACs)	C57BL/6J mice fed an HFHS diet for 20 weeks supplemented with 0.5% polymeric PACs	↓F/B., ↑*Adlercreutzia* spp.,* ↑*Roseburia* spp., ↑S24-7, ↑*Bacteroides* spp*.,* ↓*Clostridium*, ↓Lachnospiraceae, ↓*Bifidobacterium* spp.*	↑8.0-fold *A. muciniphila*	↓Dyslipidemia, ↓liver damage, ↓insulin resistance; ↓inflammation, ↓intestinal permeability	[107]
	Grape polyphenols (GP) (catechins)	HFD-induced obesity in male C57BL/6J mice fed 1% GP for 13 weeks	↓F/B, ↑*Alistipes* spp., ↑*Raouterella* spp. ↓*Lactobacillus* spp., ↓*Turicibacter* spp., ↓Lachnospiraceae, ↓Clostridiales	↑*A. muciniphila* 49% and 54.8% in cecum and feces, respectively	↓BW gain, ↓adiposity, endotoxemia, and improved glucose intolerance	[111]
	Camu-camu(CC) (galloylated PACs and ellagitannins)	HFHS-induced obesity in male C57Bl/6J fed 200 mg/kg CC for 8 weeks	↓*Lactobacillus* spp., ↑*Barnesiella* spp., ↑*Bifidobacterium* spp., ↑*Turicibacter* spp.	↑*A. muciniphila* and correlated with plasma bile acids	↓Hepatosteatosis, ↓metabolic endotoxemia, ↓glucose intolerance	[108]
	Camu-camu polyphenolic extract (CCE)	HFD-induced obesity in male C57BL/6J mice fed 200 mg/kg or 62.5 mg/kg CCE for 5 weeks	NA	↑*A. muciniphila* by 62.5 mg/kg CCE	↓Dyslipidemia, ↓BW, ↓hepatosteatosis	[109]
	Catechin-rich Zhenjiang aromatic vinegar extract (ZAVE)	Long-term alcohol consumption in ICR male mice gavaged daily with 200 or 800 mg/kg BW of ZAVE for 30 days	↓F/B, ↑Lachnospiraceae_NK4A136_group, ↑*Bacteroides* spp., ↓*Bilophila* and ↓*Butyricimonas* spp.	↑*A. muciniphila,* correlated with ↑antimicrobial peptides, ↓oxidative stress, and ↓inflammation	↓Gut inflammation, ↑IL-22, ↑Reg3g, ↓liver damage	[110]
	Green tea (GTE) (48% EGCG)	HFD-induced obesity in male C57BL/6J mice fed 2% GTE for 8 weeks	↓F/B, ↑microbial diversity, ↑Actinobacteria, ↑Coriobacteriales, ↑Turicibacterales, ↓Clostridiales, ↑B. pseudolongum*, ↑B. adolescentis*	↑*A. muciniphila* by all the tea infusions	↓Adipose inflammation, ↓metabolic endotoxemia, ↑TJPs in ileum and colon	[112]
	Green, oolong, and black teas (flavan-3-ols)	Male C57BL/6J mice fed an HFD supplemented with 45% energy from fat as food and tea infusions as drinking water for 13 weeks	↑Microbial diversity, ↑*Alistipes*, ↑Lachnospiraceae, ↑*Rikenella microfusus*, ↓*Allobaculum* spp., ↓*B. acidifaciens*, ↓*Clostridium leptum*, ↓*Parabacteroides goldsteinii*	↑*A. muciniphila* and negatively correlated with serum LBP levels	↓BW, ↓fat tissue accumulation, ↓metabolic endotoxemia, ↑lipid and glucose metabolism	[113]
**Phenolic** **Metabolites**	Urolithin A (UA) and urolithin B (UB)	Male Wistar rats fed a normal diet receiving an IP injection of UA or UB (2.5 mg/kg each) for 4 weeks	↓F/B, ↑Flavobacteriales by UA, ↓Lactobacillales and, ↓Clostridiales by UA and UB	↑*A. muciniphilia* by UA and UB	↓Serum AST	[114]

* Some species belonging to this genus or family exhibit polyphenol-degrading abilities. BW, body weight; HFD, high-fat diet; HFHS, high-fat, high-sucrose diet; IP, intraperitoneal; F/B, Firmicutes-to-Bacteroidota ratio; NA, not applicable; TJPs, tight-junction proteins; GC, goblet cells; LBP, lipid and lipopolysaccharide-binding protein.

**Table 4:** Modulation of the gut microbiota by flavonols and flavonol glycosides in animal models.

Polyphenol-Rich Foods		Experimental Design	Main Findings in the Gut Microbiota	*A. muciniphila* Modulation	Impact on Host Health	Ref.
**Flavonols**	Quercetin	HFD-induced obesity in LDLR−/− (LDL receptor-deficient) C57BL/6 mice fed 100 μg of quercetin daily for 12 weeks	↑Microbial diversity, ↑Actinobacteria, ↑Bacteroidota ↓Firmicutes, ↓*Lactobacillus*,* ↑*Bacteroides*,* ↑Parabacteroides, ↑*Ruminococcus*	↑2.0-fold *A. muciniphila*	↓BW, ↓Intestinal cholesterol, ↓oxidative stress, ↓inflammation	[131]
		HFHS-induced obesity in Wistar rats fed 30 mg/kg BW quercetin for 6 weeks	↓F/B, ↓Erysipelotrichaceae, ↓*Bacillus*, ↓*Eubacterium cylindroides*, ↑*Barnesiella*, ↑*Bacteroides dorei*, ↑*Bacteroides chinchillae*, ↑*Prevotella*	↑1-8-fold *A. muciniphila* (13.84%)	↓Insulin resistance, ↑TJPs	[132]
	Quercetin + resveratrol	HFD-induced obesity in Wistar rats fed 30 mg/kg BW quercetin, 15 mg/kg BW of resveratrol, or both for 10 weeks	↑Microbial diversity, ↑Bacteroidales_S24-7_group, ↑Ruminococca-ceae, ↑Christensenellaceae, ↓*Lachnoclostridium*, ↓*Bilophila*	↑ *A. muciniphila*	↓BW, ↓serum lipids, ↓inflammatory markers	[133]
	Quercetin (Q) + *A. muciniphila* cells	HFD-induced obesity in Wistar rats fed with 2 × 10^8^ CFU/200 µL and 37.5 mg/kg Q for 3 weeks	↑Cyanobacteria, ↑*Oscillospira* spp., ↓Actinobacteria, ↓*Lactococcus* spp., ↓*Lactobacillus* spp.,* ↓*Blautia* spp., ↓*Rothia* spp., ↓*Roseburia* spp.	↑*A. muciniphila* only when co-administered with Q. It correlated with ↓BW, lipid, and bile acid metabolism	↓BW, ↓fat mass depot, ↓NAFLD	[39]
	*Abelmoschus manihot* flowers (TFA) (Flavonol glycosides)	DSS-induced colitis in C57BL/6J mice gavaged with 125 mg/kg or 62.5 mg/kg of TFA for 7 days	↑*Gordonibacter* spp.,* ↑*Erysipelatoclostridium* spp.*,* ↓Tenericutes, ↓Proteobacteria	↑*A. muciniphila* (only by 125 mg/kg TFA), correlated with ↓gut inflammation, ↑*Muc2*, ↑barrier function	↓ Colonic inflammatory, ↓intestinal epithelial barrier dysfunction	[137]

* Some species belonging to this genus or family exhibit polyphenol-degrading abilities. Modulation relates to relative colonic or cecal abundance. BW, body weight; HFD, high-fat diet; HFHS, high-fat, high-sucrose diet; NAFLD, non-alcoholic fatty liver disease; DSS, dextran sulfate sodium; F/B, Firmicutes-to-Bacteroidota ratio; TJPs, tight-junction proteins.

**Table 5:** Modulation of the gut microbiota and *A. muciniphila* by flavones and phenolic acids in animals.

Polyphenol-Rich Foods		Experimental Design	Main Findings in the Gut Microbiota	A. *muciniphila* Modulation	Impact on Host Health	Ref.
**Flavanones**	Hesperidin	HFD-induced obesity in male C57BL/6 mice gavaged with 100 or 200 mg/kg BW hesperidin for 10 weeks	↑*Lactobacillus salivarius*, ↑Desulfovibrio _C21_c20, ↓*Helicobacter* spp.*,* ↓*B. pseudolongum* and ↓*Mucispirillum schaedleri*	Failed to change *A. muciniphila*	↓BW, ↓inflammation, ↓plasma LBP, ↑intestinal integrity	[140]
**Flavonones**	Apigenin	HFD-induced obese male C57BL/6 J mice gavaged with 50 mg/kg BW apigenin for 16 weeks	↓F/B, ↑Bacteroidaceae, ↓Erysipelotrichaceae	↑*A. muciniphila* (Akkermansiaceae)	↓Metabolic endotoxemia, ↓inflammation, ↓liver injury, ↓hepatosteatosis, ↑intestinal integrity	[144]
**Phenolic acids**	Caffeic acid (CaA)	DSS-induced colitis in female C57BL/6 mice gavaged with 1 mM CaA for 15 days	↑Microbial diversity, ↓F/B, ↑Tenericutes	↑*A. muciniphila* (25%)	↓Gut and serum inflammatory markers, ↓NF-κB signaling pathways	[149]
	Chlorogenic acid (ChA)	DSS-induced colitis in female C57BL/6 mice gavaged with 1 mM ChA for 15 days	↓F/B, ↑microbial diversity	↑*A. muciniphila* (38%)	↓Diarrhea and rectal bleeding, ↓mucin depletion, ↓gut inflammation	[148]
	Rice bran fiber-bound phenolic acids (RBDF) (p-coumaric acid, hydroxybenzoic acid, and ferulic acid)	In vitro colonic fermentation of GI-digested RBDF	↑*F. prausnitzii*, ↑*Bifidobacterium*,* ↑Lactobacillus* spp.	↑*A. muciniphila* only by the fiber-bound phenolics but not by the phenolic-free fibers	↑Antioxidant and hypoglycemic activities	[151]

* Some species belonging to this genus or family exhibit polyphenol-degrading abilities. Modulation relates to relative colonic or cecal abundance. BW, body weight; HFD, high-fat diet; HFHS, high-fat, high-sucrose diet; DSS, dextran sulfate sodium; F/B, Firmicutes-to-Bacteroidota ratio; TJPs, tight-junction proteins; GC, goblet cells; LBP, lipid- and lipopolysaccharide-binding protein.

**Table 6:** Modulation of the gut microbiota and *A. muciniphila* by stilbenes and lignans in animals.

Polyphenol-Rich Foods		Experimental Design	Main Findings in the Gut Microbiota	A. *Muciniphila* Modulation	Impact on Host Health	Ref.
**Stilbenes**	Resveratrol (RSV)	TMAO-induced atherosclerosis in ApoE−/− female C57BL/6 mice fed a chow diet with 0.4%RSV for 30 days	↑*Bacteroides*,* ↑*Lactobacillus* spp.,* ↑*Bifidobacterium* spp.,* ↓*Prevotella* spp., ↓Ruminococcaceae, ↓*Anaerotruncus* spp., ↓*Alistipes* spp., ↓Peptococcaceae	↑*A. muciniphila*	Protected against atherosclerosis, ↓gut microbial TMA production	[158]
		HFD-induced obesity in male Sprague–Dawley rats fed 100 mg/kg RSV for 6 weeks	↑Ruminococcaceae, ↑Lachnospiraceae, ↓*Desulfovibrio* spp.	↑*A. muciniphila*, correlated with endocannabinoid system modulation.	↑TJPs, ↓CB1, ↓CB2, ↓steatohepatitis, ↓gut inflammation, ↓metabolic endotoxemia	[159]
		TNBS-induced colitis in female BALB/c mice fed 100 mg/kg RSV for 5 days	↓*B. acidifaciens*, ↑*Ruminococcus gnavus*	↑4.5-fold *A. muciniphila*	Attenuated colitis, ↓gut inflammation, ↑butyrate	[160]
	Pterostilbene (Pst)	Zucker (fa/fa) rats fed a standard diet and gavaged with 15 mg/kg BW Pst for 6 weeks	↓F/B, ↑*Odoribacter splanchnicus*	↑*A. muciniphila*, correlated with ↓BW	↓BW, ↓fat mass, ↓serum insulin, ↓glucose intolerance	[162]
**Lignans**	Flaxseed (FS) (secoisolariciresinol diglucoside)	C57BL/6 mice fed a standard diet with 10% FS for 3 weeks	↑20-fold *Prevotella* spp.*,* ↑10-fold *Roseburia* spp.	↓30-fold *A. muciniphila*	↑GC, ↑Muc2, ↑RegIIIγ in the colon	[167]
	Syringaresinol (SYR)	Middle-aged male C57BL/6 mice fed 10 or 50 mg/kg BW of SYR for 10 weeks	↓F/B, ↑*Lactobacillus* spp.,* ↑*B. pseudolongum*,* ↓Bacteroidaceae*	↓*A. muciniphila* by high dose of SYR	↓LBP, ↑Foxp3+ regulatory T cells	[168]

* Some species belonging to this genus or family exhibit polyphenol-degrading abilities. Modulation relates to relative colonic or cecal abundance. BW, body weight; HFD, high-fat diet; HFHS, high-fat, high-sucrose diet; DSS, dextran sulfate sodium; F/B, Firmicutes-to-Bacteroidota ratio; TJPs, tight-junction proteins; GC, goblet cells; LBP, lipid- and lipopolysaccharide-binding protein; TMAO, trimethylamine-N-oxide; GI, gastrointestinal; TNBS, 2,4,6-trinitrobenzenesulfonic acid; TMA, trimethylamine; CB1- CB2, cannabinoid receptor type 1 and 2.

**Table 7:** Modulation of relative colonic levels of *A. muciniphila* and other bacteria by polyphenol-rich foods in human intervention trials.

Polyphenol-Rich Foods		Experimental Design	Main Findings in the Gut Microbiota	*A. muciniphila* Modulation	Impact on Host Health	Ref.
**Phenolic acids**	Oats rich in (β-glucans and polyphenol s)	Intake of 80 g of oat comprising by β-glucans (3.0 g) and polyphenols (56.8 mg) in mildly hypercholesterolemic subjects for 45 days	↑*Roseburia* spp.*,* ↑*Prevotella* spp., ↑*Paraprevotella* spp., ↑*Dialister succinatiphilus*, ↑*Roseburia hominis*, ↑*Butyrivibrio crossotus*, ↑*B. pseudocatenulatum*,* ↑*Clostridium symbiosum*, ↓*Megamonas hypermegale*, ↓*Clostridium nexile*, ↓*Roseburia inulinivorans*	↑*A. muciniphila*, correlated with ↓HDL-C	↓Dyslipidemia, ↑propionate, and ↑acetate	[171]
**Flavan-3-ols and stilbenes**	Epigallocatechin-3-gallate (EGCG) + resveratrol (RSV)	Intake of EGCG (282 mg/day) and RES (80 mg/day) in overweight and obese men and women for 12 weeks	↓Bacteroidota in men	No changes were detected in *A. muciniphila*	Improved fat oxidation in men	[172]
	Resveratrol (RSV)	Intake of 1g of RSV twice daily in obese men with MetS for 30 days	↓*Alistipes*, ↓*Collinsiella*, ↓*Christensenella*, ↓*Holdemania*, and ↓*Turicibacter* spp. in Caucasian men	↑*A. muciniphila* only in Caucasian men	Improved glucose homeostasis only in Caucasian men	[161]
	Magnolia berry Schisandra Chinensis (SCF)	Intake of 100 mL of juice twice a day containing 12 mg total phenolics and 3.34 mg total flavonoids in obese woman for 12 weeks	↑*Roseburia*, ↑*Prevotella*, ↑Bifidobacterium,* and ↑Bacteroides* spp.	↑*A. muciniphila*	↓Fat mass, ↓fasting blood glucose, ↓TG, ↓AST, and ↓ALT	[173]
**Flavanones**	Orange juice (hesperidin)	Intake of orange juice (300 mL d_−1_) for 60 days in healthy women	↑*Lactobacillus* spp.,* ↑*Ruminococcus* spp.	↑*A. muciniphila*, correlated with improved glycemia and dyslipidemia	↓Glucose intolerance, ↓insulin resistance, ↓TG	[40]
**Flavan-3-ols**	Mango (quercetin and kaempferol glycosides, gallic acid and gallotannins)	Intake of 300–400 g of mango pulp in patients with IBD for 8 weeks	↑ *L. plantarum*,* ↑*L. lactis,* ↑ L. reuteri*	No changes were detected in *A. muciniphila*	↓Plasma levels of proinflammatory cytokines, ↑butyrate	[174]
	Pomegranate juice (PJ) (Ellagitannins) and urolithin A (UA)	Intake of PJ (240 mL) for 3 weeks and UA (500 mg) for 48 h in healthy subjects	↓F/B and ↑microbial diversity, ↑Clostridiales, and ↑Ruminococcaceae in UA producers; ↑F/B and ↓in non-UA producers	↑*A. muciniphilia* in high UA producers	6.0-fold ↑plasma UA glucuronide after UA intake compared to PJ	[42]
	Pomegranate extract	Intake of POM (1 g/day) in healthy volunteers for 4 weeks categorized as urolithin A (UA) producers and non-producers	↑Actinobacteria, ↓Firmicutes, ↑*Butyrivibrio* spp., ↑*Enterobacter* spp., ↑*Escherichia* spp., ↑*Lactobacillus* spp.,* ↑*Prevotella* spp., ↓*Collinsella* spp. in UA producers	47-fold ↑*A. muciniphilia* in UA producers	↑Plasma UA glucuronide	[43,52]

* Some species belonging to this genus or family exhibit polyphenol-degrading abilities. F/B, Firmicutes-to-Bacteroidota ratio; IBD, inflammatory bowel disease; TG, triglycerides; AST, aspartate aminotransferase; ALT, alanine aminotransferase.
